# A review of the auditory-gut-brain axis

**DOI:** 10.3389/fnins.2023.1183694

**Published:** 2023-08-03

**Authors:** Amy S. Graham, Benneth Ben-Azu, Marie-Ève Tremblay, Peter Torre, Marjanne Senekal, Barbara Laughton, Andre van der Kouwe, Marcin Jankiewicz, Mamadou Kaba, Martha J. Holmes

**Affiliations:** ^1^Imaging Sciences, Neuroscience Institute, University of Cape Town, Cape Town, South Africa; ^2^Department of Human Biology, Division of Biomedical Engineering, University of Cape Town, Cape Town, South Africa; ^3^Division of Medical Sciences, University of Victoria, Victoria, BC, Canada; ^4^Department of Pharmacology, Faculty of Basic Medical Sciences, College of Health Sciences, Delta State University, Abraka, Delta State, Nigeria; ^5^Département de Médecine Moléculaire, Université Laval, Québec City, QC, Canada; ^6^Axe Neurosciences, Centre de Recherche du CHU de Québec, Université Laval, Quebec City, QC, Canada; ^7^Neurology and Neurosurgery Department, McGill University, Montreal, QC, Canada; ^8^Department of Biochemistry and Molecular Biology, University of British Columbia, Vancouver, BC, Canada; ^9^Centre for Advanced Materials and Related Technology (CAMTEC), University of Victoria, Victoria, BC, Canada; ^10^Institute for Aging and Lifelong Health, University of Victoria, Victoria, BC, Canada; ^11^School of Speech, Language, and Hearing Sciences, San Diego State University, San Diego, CA, United States; ^12^Department of Human Biology, Division of Physiological Sciences, University of Cape Town, Cape Town, South Africa; ^13^Family Clinical Research Unit, Department of Pediatrics and Child Health, Stellenbosch University, Cape Town, South Africa; ^14^Department of Radiology, Athinoula A. Martinos Center for Biomedical Imaging, Massachusetts General Hospital, Boston, MA, United States; ^15^Department of Radiology, Harvard Medical School, Boston, MA, United States; ^16^Department of Pathology, Division of Medical Microbiology, University of Cape Town, Cape Town, South Africa; ^17^Department of Biomedical Physiology and Kinesiology, Simon Fraser University, Burnaby, BC, Canada; ^18^ImageTech, Simon Fraser University, Surrey, BC, Canada

**Keywords:** gut-brain axis, microbiome, auditory system, hearing loss, noise

## Abstract

Hearing loss places a substantial burden on medical resources across the world and impacts quality of life for those affected. Further, it can occur peripherally and/or centrally. With many possible causes of hearing loss, there is scope for investigating the underlying mechanisms involved. Various signaling pathways connecting gut microbes and the brain (the gut-brain axis) have been identified and well established in a variety of diseases and disorders. However, the role of these pathways in providing links to other parts of the body has not been explored in much depth. Therefore, the aim of this review is to explore potential underlying mechanisms that connect the auditory system to the gut-brain axis. Using select keywords in PubMed, and additional hand-searching in google scholar, relevant studies were identified. In this review we summarize the key players in the auditory-gut-brain axis under four subheadings: anatomical, extracellular, immune and dietary. Firstly, we identify important anatomical structures in the auditory-gut-brain axis, particularly highlighting a direct connection provided by the vagus nerve. Leading on from this we discuss several extracellular signaling pathways which might connect the ear, gut and brain. A link is established between inflammatory responses in the ear and gut microbiome-altering interventions, highlighting a contribution of the immune system. Finally, we discuss the contribution of diet to the auditory-gut-brain axis. Based on the reviewed literature, we propose numerous possible key players connecting the auditory system to the gut-brain axis. In the future, a more thorough investigation of these key players in animal models and human research may provide insight and assist in developing effective interventions for treating hearing loss.

## 1. Introduction

### 1.1. Problem statement

Hearing loss affects more than 20% of the global population, with severity ranging between moderate and complete loss of hearing in over 5% of people worldwide (Haile et al., 2021). Although the elderly (>70 years) and children below the age of 5 years are the most at-risk populations (Haile et al., 2021), hearing loss can occur at different ages and for various reasons (Zahnert, 2011). Hearing loss in adults and children can vary in severity (Gopinath et al., 2012; Tomblin et al., 2015) and can occur in only one or both ears (Oh et al., 2007; Fitzpatrick et al., 2010; Golub et al., 2018). There are four main categories of hearing loss – conductive, sensorineural, mixed and central hearing loss (Isaacson and Vora, 2003; Zahnert, 2011).

Conductive hearing loss occurs when the conduction of sound from the outer ear through the middle ear to the cochlear, within the inner ear, is disrupted (Carpena and Lee, 2018). This can result from damage to middle ear structures, such as the tympanic membrane, mastoid or ossicular chain (Kim et al., 2019). Infections of the middle ear (Graydon et al., 2017) or abnormal growths around the tympanic membrane (Martins et al., 2012) can also lead to conductive hearing loss.

Sensorineural hearing loss (SNHL) results from signals being incorrectly relayed from the cochlea to the brain – either due to vibration signals not being correctly converted into electrical signals within the cochlea, or nerve injury (Carpena and Lee, 2018). Congenital SNHL can be genetically inherited or arise from viral infections such as congenital cytomegalovirus infection (van Beeck Calkoen et al., 2019). The toxic effects of chemotherapeutic drugs and antibiotics within the ear may also cause SNHL (Wang et al., 2015a; Garinis et al., 2017). Mixed hearing loss involves a combination of conductive hearing loss and SNHL.

Finally, central hearing loss involves disruption to the central auditory system – which begins with the auditory nerve exiting the cochlea and continues through to the auditory cortex of the brain (Zahnert, 2011). Central hearing loss and processing disorders of the central auditory system have many possible causes, including brain lesions (Jerger, 1987), meningitis infections (Kihara et al., 2012), brain injuries (Bergemalm and Lyxell, 2005; Oleksiak et al., 2012), strokes (Bamiou et al., 2012; Fujioka et al., 2020; Lachowska et al., 2021), multiple sclerosis (MS) (Matas et al., 2010; Valadbeigi et al., 2014), heavy metal poisoning (Dutra et al., 2010; Alvarenga et al., 2015; Castellanos, 2016), prenatal hypoxia (Jiang et al., 2008) and can also occur with age (Kim and Chung, 2013).

Many studies have investigated the underlying mechanisms responsible for hearing loss (Willems, 2000; Li et al., 2018; Chen et al., 2019; Uchida et al., 2019) and have identified interventions that can prevent or delay its onset. However, there is still much to be understood about the auditory system, how it interacts with other parts of the body and how the burden of hearing loss can be addressed globally.

### 1.2. The auditory system

**Figure 1:**
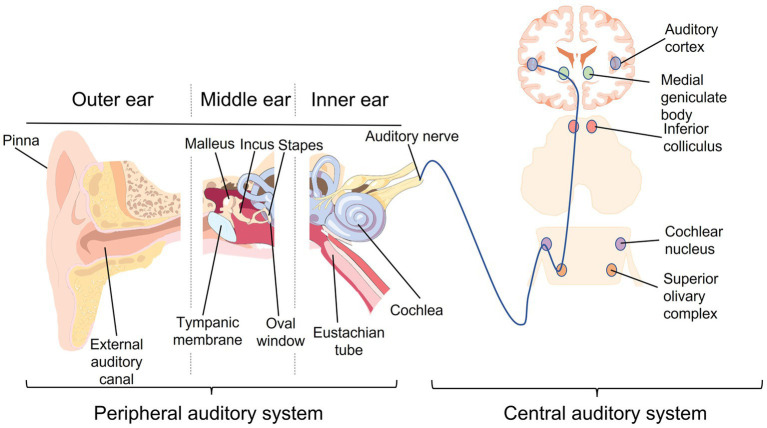
A visual summary of the main components of the peripheral and central auditory systems. Inspired by previous figures of auditory anatomy (Cope et al., 2015; Nipa et al., 2020). A component of the figure used the following online resource which has Creative Commons CC0: https://openclipart.org/detail/281964/ear-anatomy by GDJ; Creative Commons CC0.

The auditory system can be divided into two clear components (Figure 1). The peripheral auditory system includes the structures of the outer, middle and inner ear where sound is received and converted into electrical signals. The central auditory system contributes to transportation of electrical auditory signals from the cochlea to the brain, where they are processed.

#### 1.2.1. The peripheral auditory system

Auditory signals are directed into the ear canal by the pinna and travel through to the tympanic membrane, the lateral border of the middle ear (Alberti, 2001). The functions of the outer ear include filtering sound according to frequency, and also amplification of auditory signals (Sundar et al., 2021). The outer ear also plays an important role in directional hearing whereby, based on the frequency of sound and the timing at which it reaches each ear, the direction of the source can be determined (Fischer and Schäfer, 1991).

The middle ear spans from the tympanic membrane at the lateral side to the oval window of the cochlea at its medial end (Alberti, 2001). There are three important bones involved in the transmission of sound through the middle ear: the malleus, incus and stapes (Alberti, 2001; Sundar et al., 2021). Sound is transmitted from the tympanic membrane and along these bones in the form of mechanical signals (Aernouts et al., 2012; Rusinek, 2021). Amplification of sound also takes place in the middle ear – this is vital due to the transfer of sound from air to fluid as it reaches the cochlea of the inner ear, upon which there is resistance due to an increase in density of the medium the sound is traveling through (Sundar et al., 2021). This concept has been termed “impedance matching” (Parent and Allen, 2010). The middle ear is also where connections to the nose and respiratory system occur through the eustachian tube and mastoid air cells, respectively (Alberti, 2001; Sundar et al., 2021).

In the inner ear, there are a number of important structures, including the vestibular labyrinth for balance and the membranous labyrinth within the cochlea (Alberti, 2001). Mechanical signals are converted to electrical signals here. As the stapes vibrates, this causes the fluid surrounding the membranous labyrinth also to vibrate (Alberti, 2001). The vibrations in fluid are converted into electrical action potentials, by which auditory signals are transmitted along the auditory nerve to the brain (Sundar et al., 2021).

#### 1.2.2. The central auditory system

The auditory nerve marks the beginning of the central auditory system. Here the auditory system and brain intersect. Electrical signals are carried from the cochlea to the cochlear nucleus in the brainstem via this nerve (Celesia and Hickok, 2015). The signal is transmitted to the superior olivary complex, passed to the inferior colliculus, and then relayed to the medial geniculate body (Celesia and Hickok, 2015). Finally auditory signals travel to the auditory cortex where the main signals are processed in the primary auditory cortex, while processing more detailed sound also requires the activity of secondary cortex regions (Patterson et al., 2002). White matter tracts connect structures of the central auditory system (Javad et al., 2014). Damage or disruptions to the peripheral auditory system can lead to detrimental effects downstream in the central auditory system (Syka, 2002; Butler and Lomber, 2013).

### 1.3. The gut-brain axis

The gut-brain axis has become a major research focus as more studies have found links between the brain and microbial populations of the gut. Disruption to the gut microbiome composition, commonly termed ‘gut dysbiosis’, has been observed in various diseases and disorders of the brain (Tremlett et al., 2017; Vogt et al., 2017; Haran et al., 2019; Romano et al., 2021). Disorders in the gut can also impact the brain and behavior (Gracie et al., 2017; van Langenberg et al., 2017; Ng et al., 2018).

A number of mechanisms are believed to contribute to the bidirectional signaling between the brain and gut (Cryan et al., 2019). Various studies in animals and humans have identified the vagus nerve, hypothalamic–pituitary–adrenal (HPA) axis, the immune system, neurotransmitters and bacterial metabolites as probable regulators of gut-brain axis signaling (Ait-Belgnaoui et al., 2012; Iven et al., 2019; Lin et al., 2019; Strandwitz et al., 2019; Zhang et al., 2020; Kong et al., 2021). However, there is still much to be understood about these various signaling pathways and the roles they play in health and disease.

### 1.4. The gut-auditory axis

Various genes have been implicated in the development of the gut and ear (Bitner-Glindzicz et al., 2000; Stanchina et al., 2006; Crawley et al., 2014). As such, mutations in these genes can lead to disruptions in the function of the gut and can also impact the ear and hearing. For instance, a mutation in the ‘rearranged during transfer’ (*RET*) gene during the embryonic period is commonly associated with Hirschsprung’s disease in humans (Fadista et al., 2018). This gene is required for the establishment of the enteric nervous system (ENS) during development (Luesma et al., 2014). A study investigating the role of this gene in Hirschsprung’s disease showed that mutations in the *c-RET* and *c-Ret* genes also led to hearing loss in human and rodent models, respectively, (Ohgami et al., 2010). A more recent animal study has shown that mutations in this gene may additionally lead to underdevelopment of the cerebellum in the brain (Ohgami et al., 2021).

In mice with mutations in genes coding for SRY-related HMG-box 10 (*Sox10*), endothelin-3 (*Edn3*) and endothelin-B receptor (*Ednrb*), excessive dilation of the colon and abnormal ENS development have also been previously observed (Stanchina et al., 2006). The authors suggested that terminal differentiation and apoptosis of neural crest cells may lead to fewer of these cells entering the gut regions during development – ultimately causing abnormal gut development which can shorten the lifespan of mice. Additionally, mutations in these genes were shown to reduce the number of melanocytes in the ear, where they are required for cochlea function (Stanchina et al., 2006). Furthermore, in mice with a dominant *Sox10* mutation and double mutations in the *Ednrb* gene, there were absolutely no melanocytes present (Stanchina et al., 2006). Human research has also identified the importance of melanocytes (and the melanin pigment they carry) in ear development, while SOX10 is commonly used as a cell type marker for melanocytes (van Beelen et al., 2020), which reinforces the connection between ENS and hearing activity.

Mutations in the Usher type 1C (*USH1C* in humans, *Ush1c* in animals) gene likewise impact the ear and gut (Bitner-Glindzicz et al., 2000; Crawley et al., 2014). Congenital SNHL and disruptions in gut function are reported in individuals with Usher type 1 syndrome (Bitner-Glindzicz et al., 2000). *In vitro* research and animal studies suggest that protein–protein interactions that maintain structure and stability between stereocilia of the ear and between microvilli of the gut, are impacted in Usher syndrome (Verpy et al., 2000; Siemens et al., 2002; Crawley et al., 2014). This is due to *USH1C* genes encoding harmonin, a protein that is important for creating protein complexes with cadherins, which if mutated leads to disruptions to the arrangement of microvilli and stereocilia (Siemens et al., 2002; Crawley et al., 2014). Further, the organization of hair cells has been found to be affected in a small study of mice with mutations in *Ush1c,* and these mice did not respond at typical thresholds used for auditory brainstem response tests *–* while wild type mice had normal auditory brainstem response thresholds (Lentz et al., 2010). These findings reinforce what has been found in other studies regarding the role of *Ush1c* in arrangement of hair cells, and it indicates that not only the peripheral auditory system is affected, but that the central auditory system is also impacted in Usher syndrome.

Therefore, these genetic disorders provide evidence of a link between the gut and the ear, providing the third arm in this 3-way relationship between the ear, gut and brain.

Several recent reviews have begun to touch on aspects of the auditory-gut-brain axis (Kociszewska and Vlajkovic, 2022; Megantara et al., 2022) or ear-gut axis (Denton et al., 2022), focusing largely on the role of the immune system and inflammation in establishing these connections. Furthermore, they address how neurotransmitters, metabolic activity and disruptions to protective barriers may play a role in diseases and disorders through which we can observe interactions between the ear, gut and brain.

### 1.5. Aim

Although the gut-brain axis is a field of research that is rapidly gaining traction, there are many aspects that have yet to be explored in depth. One such aspect is the link between the auditory system and gut-brain axis. While evidence for a gut-ear axis is starting to be explored in conjunction with links to inflammation of the brain, there are many potential pathways of communication between the auditory system and gut-brain axis which are yet to be considered. As hearing loss contributes substantially to the burden of disease across the world, investigating a potential link between the auditory system and gut-brain axis may contribute valuable insight for treating diseases or disorders related to the ear and/or hearing. In exploring this, we might also build on mechanisms of hearing loss and on knowledge of underlying signaling pathways within the human body.

The ear has a microbiome of its own, which would be expected to communicate and interact with microbiomes located in other parts of the body, including the gut. Further, there are anatomical and physiological mechanisms which play a prominent role in both the auditory system and gut-brain axis (Klarer et al., 2014; Kondo et al., 2020). The goal of this review is to identify studies which show a link between the auditory system and gut-brain axis, and to outline putative mechanisms by which these systems could interact.

## 2. Methodology

A scoping review of literature was carried out to identify papers of interest that could address the important topics in this field. PubMed was searched using various sets of keywords (Table 1), and additional hand-searching was done in Google Scholar and Science Direct. We summarize how many results were obtained using each set of keywords, as well as identifying which of these papers were ultimately included in the review.

**Table 1:** PubMed keywords used in this review.

Review Section	Keywords used in PubMed	Number of papers found with keyword search	Results from search included in this review
Section 3	(“gut-brain axis” [All Fields] OR “gut-brain-axis” [All Fields] OR “microbiota-gut-brain axis”[All Fields] OR “microbiota-gut-brain-axis”[All Fields] OR “microbiome-gut-brain axis”[All Fields] OR “gut-microbiome-brain axis”) AND (“auditory” [All Fields] OR “ear” [All Fields] OR “ear” [MeSH Terms] OR “hearing” [All Fields] OR “hearing” [MeSH Terms] OR “noise”[MeSH Terms] OR “noise”[All Fields])	13	Chi et al. (2021), Cui et al. (2018), Hickey et al. (2020), Klarer et al. (2014)
Section 3	((“gut”[All Fields] OR “stomach”[MeSH Terms] OR “stomach”[All Fields] OR”gastrointestinal”[All Fields] OR “intestinal”[All Fields] OR “intestines”[MeSH Terms] OR “intestines”[All Fields]) AND (“disease”[MeSH Terms] OR “disease”[All Fields] OR “disorder”[All Fields] OR “disorders”[All Fields])) AND (“ear”[MeSH Terms] OR “ear”[All Fields] OR “auditory”[All Fields] OR “hearing”[MeSH Terms] OR “hearing”[All Fields] OR “deafness”[MeSH Terms] OR “deafness”[All Fields])	1,507	Bitner-Glindzicz et al. (2000), Fang et al. (2020), Kondo et al. (2020), Leggio et al. (2007), Nadeem et al. (2017), Oike et al. (2016), Sonoyama et al. (2011), Stanchina et al. (2006)
Section 4.1.1	(“vagus nerve”[MeSH Terms] OR “vagus nerve”[All Fields]) AND (“ear”[MeSH Terms] OR “ear”[All Fields] OR “auditory”[All Fields] OR “hearing”[MeSH Terms] OR “hearing”[All Fields] OR “deafness”[MeSH Terms] OR “deafness”[All Fields])	776	Baig et al. (2019), Frangos et al. (2015), Klarer et al. (2014), Lehtimäki et al. (2013), Ventureyra (2000), Rong et al. (2016), Ylikoski et al. (2020)
Section 4.1.2	((“nose”[MeSH Terms] OR “nose”[All Fields] OR “nasal”[All Fields] OR “nasopharynx”[MeSH Terms] OR “nasopharynx”[All Fields] OR “nasopharyngeal”[All Fields] OR “pharyngeals”[All Fields] OR “pharynges”[All Fields] OR “pharynx”[MeSH Terms] OR “pharynx”[All Fields] OR “pharyngeal”[All Fields] OR “nostril”[All Fields] OR “nostrils”[All Fields] OR “nare”[All Fields] OR “nares”[All Fields]) AND (“ear”[MeSH Terms] OR “ear”[All Fields])) AND (“microbiota”[MeSH Terms] OR “microbiota”[All Fields] OR “microbiome”[All Fields] OR “microbiomes”[All Fields] OR microflora OR metagenome OR virome OR mycobiome)	114	Brugger et al. (2019), Chan et al. (2017), Coleman et al. (2021), Frank et al. (2021), Johnston et al. (2019), Lee et al. (2021), Man et al. (2019), Xu et al. (2019), Xu J. et al. (2020)
Section 4.1.2	(“mouth”[MeSH Terms] OR “mouth”[All Fields] OR “oral”[All Fields]) AND (“ear”[MeSH Terms] OR “ear”[All Fields])) AND (“microbiota”[MeSH Terms] OR “microbiota”[All Fields] OR “microbiome”[All Fields] OR “microbiomes”[All Fields] OR microflora OR metagenome OR virome OR mycobiome)	41	Brugger et al. (2019), Frank et al. (2021), Lee et al. (2021), Man et al. (2019)
Section 4.1.3	(labyrinth OR motion sickness) AND (“ear”[MeSH Terms] OR “ear”[All Fields] OR “auditory”[All Fields] OR “hearing”[MeSH Terms] OR “hearing”[All Fields] OR “deafness”[MeSH Terms] OR “deafness”[All Fields]) AND (“gut”[All Fields] OR “stomach”[MeSH Terms] OR “stomach”[All Fields] OR”gastrointestinal”[All Fields] OR “intestinal”[All Fields] OR “intestines”[MeSH Terms] OR “intestines”[All Fields])	352	Kondo et al. (2020), Stanchina et al. (2006), Yates et al. (2014)
Section 4.2.1	(“HPA”[All Fields] OR “hypothalamic–pituitary–adrenal”[All Fields]) AND (“ear”[MeSH Terms] OR “ear”[All Fields] OR “auditory”[All Fields] OR “hearing”[MeSH Terms] OR “hearing”[All Fields] OR “deafness”[MeSH Terms] OR “deafness”[All Fields])	183	Graham and Vetter (2011), Newsom et al. (2020), Tahera et al. (2007)
Section 4.2.2	((“neurotransmitter agents”[MeSH Terms] OR “neurotransmitter agents”[All Fields] OR “neurotransmitter”[All Fields] OR “neurotransmitters”[All Fields]) AND (“brain”[MeSH Terms] OR “brain”[All Fields])) AND (“ear”[MeSH Terms] OR “ear”[All Fields] OR “auditory”[All Fields] OR “hearing”[MeSH Terms] OR “hearing”[All Fields] OR “deafness”[MeSH Terms] OR “deafness”[All Fields])	5,120	Elgoyhen et al. (2009), Gawron et al. (2004), Jougleux et al. (2011), Klarer et al. (2014), Schmäl (2013), Sedley et al. (2015), Zhao et al. (2009)
Section 4.2.3	(“endocannabinoids”[MeSH Terms] OR “endocannabinoids”[All Fields] OR “endocannabinoid”[All Fields]) AND (“ear”[MeSH Terms] OR “ear”[All Fields] OR “auditory”[All Fields] OR “hearing”[MeSH Terms] OR “hearing”[All Fields] OR “deafness”[MeSH Terms] OR “deafness”[All Fields])	101	Ghosh et al. (2018), Newsom et al. (2020), Valdés-Baizabal et al. (2017), Zhao et al. (2009)
Section 4.2.4	((“bacteria”[MeSH Terms] OR “bacteria”[All Fields] OR “bacterial”[All Fields] OR “microbes”[All Fields] OR “microbial”[All Fields]) AND (“metabolite”[All Fields] OR “metabolites”[All Fields] OR “SCFA”[All Fields] OR “short chain fatty acids”[All Fields])) AND (“ear”[MeSH Terms] OR “ear”[All Fields] OR “auditory”[All Fields] OR “hearing”[MeSH Terms] OR “hearing”[All Fields] OR “deafness”[MeSH Terms] OR “deafness”[All Fields])	92	Fang et al. (2020), Kondo et al. (2020), Nadeem et al. (2017), Oike et al. (2016), Saha et al. (2016),
Section 4.3	(“immune”[All Fields] OR “autoimmune”[All Fields] OR “immunity”[All Fields] OR “autoimmunity”[All Fields] OR “inflammation”[MeSH Terms] OR “inflammation” [All Fields] OR “inflammatory”[All Fields] OR “cytokines”[MeSH Terms] OR “cytokines”[All Fields]) AND (“ear”[MeSH Terms] OR “ear”[All Fields] OR “auditory”[All Fields] OR “hearing”[MeSH Terms] OR “hearing”[All Fields] OR “deafness”[MeSH Terms] OR “deafness”[All Fields]) AND (“gut”[All Fields] OR “stomach”[MeSH Terms] OR “stomach”[All Fields] OR”gastrointestinal”[All Fields] OR “intestinal”[All Fields] OR “intestines”[MeSH Terms] OR “intestines”[All Fields])	634	Fang et al. (2020), Hickey et al. (2020), Kang and Im (2020), Leggio et al. (2007), Nadeem et al. (2017), Saha et al. (2016), Sonoyama et al. (2011)
Section 4.4.1	(“probiotics”[MeSH Terms] OR “probiotics”[All Fields] OR “probiotic”[All Fields]) AND (“ear”[MeSH Terms] OR “ear”[All Fields] OR “auditory”[All Fields] OR “hearing”[MeSH Terms] OR “hearing”[All Fields] OR “deafness”[MeSH Terms] OR “deafness”[All Fields])	101	Fang et al. (2020), Oike et al. (2016)
Section 4.4.2	(“choline”[MeSH Terms] OR “choline”[All Fields]) AND (“ear”[MeSH Terms] OR “ear”[All Fields] OR “auditory”[All Fields] OR “hearing”[MeSH Terms] OR “hearing”[All Fields] OR “deafness”[MeSH Terms] OR “deafness”[All Fields])	644	Cheng et al. (2008), Freedman et al. (2019), Na et al. (2021), Nair et al. (2004), Sedley et al. (2015), Stevens et al. (2008)
Section 4.4.3	(“iron”[MeSH Terms] OR “iron”[All Fields]) AND (“anaemia”[All Fields] OR “anemia”[MeSH Terms] OR “anemia”[All Fields]) AND (“ear”[MeSH Terms] OR “ear”[All Fields] OR “auditory”[All Fields] OR “hearing”[MeSH Terms] OR “hearing”[All Fields] OR “deafness”[MeSH Terms] OR “deafness”[All Fields])	184	Amin et al. (2013), Golz et al. (2001), Jougleux et al. (2011), Schieffer et al. (2017a), Schieffer et al. (2017b), Sundagumaran and Seethapathy (2019), Sundagumaran and Seethapathy (2020), Yu et al. (2014)
Section 4.4.4	(“vitamin b12”[All Fields] OR vitamin b12[MeSH Terms] OR “cobalamin”[All Fields] OR cobalamin[MeSH Terms]) AND (“ear”[MeSH Terms] OR “ear”[All Fields] OR “auditory”[All Fields] OR “hearing”[MeSH Terms] OR “hearing”[All Fields] OR “deafness”[MeSH Terms] OR “deafness”[All Fields])	188	Houston et al. (1999), Kisli and Saçmacı (2019), Péneau et al. (2013), Singh et al. (2016)

## 3. Evidence of an auditory-gut-brain axis connection

Many of the key players that connect the gut microbiome and the brain may also provide a connection with the auditory system – here referred to as the auditory-gut-brain axis. An early study of patients with inflammatory bowel syndrome found differences in the processing of auditory signals compared to healthy individuals, particularly in the frontal lobe (Blomhoff et al., 2000). Since then, several research papers have provided additional evidence of connections between the ear, gut and brain.

A number of studies in animals have shown a plausible link between the auditory system and gut-brain axis. Following Pavlovian conditioning, in which rats were exposed to auditory stimuli prior to being shocked, rats in which afferent vagus nerve connections were surgically severed displayed a heightened fear response to auditory prompts, compared to rats that experienced sham procedures (Klarer et al., 2014). This indicates that the vagus nerve – an important component of gut-brain axis signaling – plays an important role in regulating or diminishing fear following noise conditioning.

Chronic exposure to noise has been shown to disrupt the gut microbiome composition and result in amyloid-β build-up and cognitive decline in mice (Cui et al., 2018). Amyloid-β is a key marker associated with the development of Alzheimer’s disease, the most prevalent neurodegenerative disease (Ferreira et al., 2015). Permeability of the intestine was also found to be impacted in noise-exposed mice, while the integrity of the blood–brain barrier (BBB) tight junctions was compromised (Cui et al., 2018). Further, mice exposed to chronic noise showed increased inflammatory markers in their blood [inducible nitric oxide synthase, nuclear factor κB and interleukin (IL)-6] and alterations to microbiome functionality that are indicative of greater oxidative stress (Chi et al., 2021). Probiotic treatment has been shown to successfully treat behavioral changes resulting from noise-induced stress during fetal development in mice (Hadizadeh et al., 2019). Collectively, these findings reveal that noise can impact the gut-brain axis and they indicate a connection to the auditory system through multiple signaling pathways.

In another mouse model study investigating age-related hearing loss, ingestion of the H61 strain of heat-killed *Lactococcus lactis* improved hearing outcomes – as revealed by a lower auditory signal intensity required to generate a response in the brainstem (Oike et al., 2016). This study provided evidence that the central auditory system can be affected by altering the microbial populations in the gut. In mice provided with a prebiotic diet with short chain fatty acid (SCFA) production properties – such as a fructo-oligosaccharide diet – up-regulations of brain-derived neurotrophic factor (BDNF) and SCFA receptor gene expression were measured within the inner ear (Kondo et al., 2020). As alterations to the gut microbiome composition were also observed by Kondo et al. (2020), this would indicate that diet-driven shifts in the microbiome can impact the ear on a gene expression level. More specifically, these shifts impacted BDNF expression, which is required for the survival of afferent neurons that transmit sensory signals to the central auditory system (Chacko et al., 2017).

A human study in one month-old infants using electroencephalography (EEG) has shown a link between antibiotic usage (ampicillin and gentamicin) and the response to auditory stimuli (Hickey et al., 2020). While infants typically have greater EEG signals in response to their mothers’ voices compared to the voice of a stranger, infants who received antibiotics showed a converse response (Hickey et al., 2020). In infants who received antibiotics, EEG signals in response to the voice of a stranger were lower in the frontal and central scalp regions, which are involved in processing auditory memory (deRegnier et al., 2000; Hickey et al., 2020). However, the amplitude reduction was even more drastic in response to hearing their own mothers’ voices (Hickey et al., 2020). This indicates that treatment with ampicillin and gentamicin – which were found in other research to alter the infant gut microbiome composition (Fouhy et al., 2012) and have toxic effects on the nervous system (Grill and Maganti, 2011) – may impact the ability of infants to remember and process sound.

Finally, studies have shown that the gut microbiome can impact the central auditory system at a cognitive level. In a study of individuals with HIV, probiotic treatment over a period of 6 months was found to significantly improve both the ability to process auditory signals and auditory memory (Ceccarelli et al., 2017). This was seen by improved Rey Auditory Verbal Learning Test outcomes in these individuals compared to their scores prior to probiotic treatment, and also compared to control individuals with HIV who did not receive probiotics over the intervention period (Ceccarelli et al., 2017). In another study by Kort et al. (2021), children assessed with the Bayley Scales of Infant and Toddler Development were found to have a relationship between gut microbiome composition and language development. This study found that a higher abundance of *Coprococcus eutactus*, a producer of the short-chain fatty acid butyrate, was associated with better language outcomes (Kort et al., 2021).

## 4. Key players that connect the auditory system, gut microbiome, and brain

There are numerous signaling pathways through which the gut microbiota and brain are believed to communicate with each other (Long-Smith et al., 2020; Margolis et al., 2021). By briefly outlining the role of various mechanisms in the gut-brain axis, we aim to provide evidence for their contribution to the auditory-gut and auditory-brain axes based on animal and human research. Ultimately, we aim to identify key players of communication in the auditory-gut-brain axis. These will be discussed under four sub-headings: anatomical, extracellular, immune system and dietary mechanisms.

### 4.1. Anatomical mechanisms

#### 4.1.1. The vagus nerve

The vagus nerve is a signaling pathway that has been clearly implicated in the gut-brain axis. It connects the medulla oblongata in the brainstem to the stomach and intestines (Breit et al., 2018). The vagus nerve plays an important role in the autonomic nervous system, regulating unconscious activity of the digestive system and various other organs of the body (de Lartigue, 2016; Breit et al., 2018). Of the vagus nerve fibers that connect the brain and gut, ~10–20% send signals from the brain to the gut while the remaining ~80–90% carry signals up from the gut to the brain (Breit et al., 2018).

Research has shown that severing the sub-diaphragmatic branch of the vagus nerve in mice changes the response to a challenge with the bacterial endotoxin component lipopolysaccharide (LPS) (Zhang et al., 2020). Peripheral introduction of LPS may cause behavioral changes through immune cell reactivity, including release of pro-inflammatory cytokines (Dantzer et al., 2008), indicating that disruptions to the gut barrier may have a neurological impact. Zhang et al. (2020) showed that while composition of the microbiome, spleen (a large immune organ) weight and behavior could be impacted by LPS, surgical severance of the sub-diaphragmatic vagus nerve branch prevented these effects of LPS. Severing this nerve normalized cytokine levels and bacterial composition (Zhang et al., 2020).

In addition to the branches of the vagus nerve that connect the gut and brain, there are several other branches. This includes the auricular branch, which provides a connection to various structures of the external ear (Frangos et al., 2015). Providing electrical stimulation through the vagus nerve is a technique that has been used for many years as a non-invasive means of treating patients with epileptic seizures (Elger et al., 2000; Ventureyra, 2000). More recently, stimulation of the auricular branch has been identified as a promising treatment in a number of neurological disorders and injuries, including major depressive disorder (Rong et al., 2016) and stroke (Baig et al., 2019). Animal research also indicates a possible therapeutic application for traumatic brain injuries (Pruitt et al., 2016), among other disease conditions. In a small cohort of adult females with inflammatory bowel syndrome, stimulation of the auricular branch of the vagus nerve was found to provide pain relief and to ease the severity of symptoms (Mion et al., 2020). The potential for auricular branch stimulation in treatment of tinnitus – a disease characterized by humming or ringing within the ears that could sometimes be caused by infection, brain damage or drug-induced ototoxicity – has also been established, with studies showing some alleviation of tinnitus-related stress and a reduction in tinnitus-related symptoms (Lehtimäki et al., 2013; Ylikoski et al., 2020). Moreover, by exploring the relationship between electrical activity of the gut and functional connectivity of the brain, auricular branch stimulation has recently been shown to reinforce the bidirectional connection between the gut and brain – specifically with regard to appetite (Müller et al., 2022).

Together these results provide evidence for an anatomical connection between the ear, gut and brain. Furthermore, they show that stimulating the auricular branch of the vagus nerve, from the external ear, can be used to treat disorders in all three parts of the body including depression, gut dysbiosis and tinnitus.

**Figure 2:**
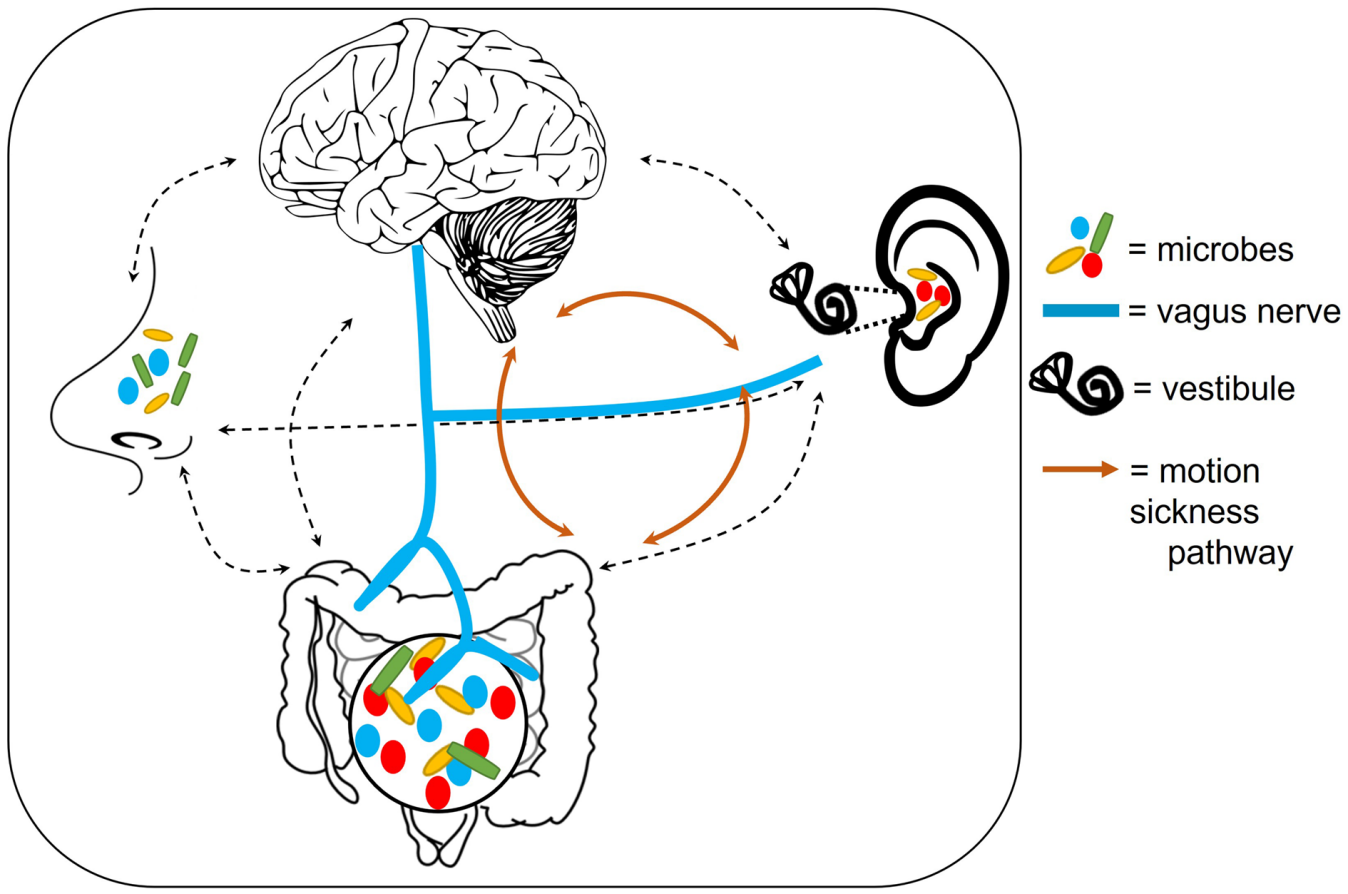
Outline of potential anatomical mechanisms that could connect the auditory-gut-brain axis. These include the vagus nerve, the nasal/oral microbiome and the motion sickness pathway. Dotted lines indicate possible pathways of microbe communication. Components of this figure used the following online resources which all have Creative Commons CC0: https://openclipart.org/detail/35791/brain-01 by rejon; https://openclipart.org/detail/269019/ear by CCX; https://www.needpix.com/photo/169098/intestines-bowel-guts-intestinal-gastrointestinal-digestive-system-abdominal-biology-science; https://openclipart.org/detail/184612/nose by Frankes.

Finally, as previously mentioned, in rats exposed to Pavlovian fear conditioning, the vagus nerve plays an important role in determining the ability to modulate the pre-emptive fear responses to auditory prompts (Klarer et al., 2014). Klarer et al. (2014) found that with every round of auditory prompts followed by shock stimuli, the fear responses dissipated more quickly in control rats compared to rats with a severed vagus nerve. As such, an auditory-gut-brain link is established by the vagus nerve (Figure 2).

#### 4.1.2. Oral, nasal, and respiratory tract microbiomes

The oral intake of probiotics can be used to treat ear infections such as otitis media (Rautava et al., 2008; Di Pierro et al., 2016). In addition, the introduction of probiotics in the form of a nasal spray – which can act via the eustachian tube – can allow otitis media to be successfully treated (Roos et al., 2001). The eustachian tube provides a connection from the nasopharynx to the middle ear (Alberti, 2001). Therefore, there is a possible link between the ear microbiome and oral, nasal or respiratory microbiomes.

Several reviews and research papers have looked at the relationship between the microbiome of the gut and those of the nasopharynx, mouth and lungs (Budden et al., 2017; Iwauchi et al., 2019; Benahmed et al., 2020; Thapa et al., 2020). A study by Budden et al. (2017) showed that the immune system may be a mechanism of communication between the lung and gut microbiomes. Thapa et al. (2020) provided evidence that antibiotics may alter both the nasopharyngeal and gut microbiomes. Disruptions to each of these microbiomes have also been suggested to contribute to brain disorders, such as Alzheimer’s and Parkinson’s diseases (Bell et al., 2019). Together these findings indicate that the microbiomes across the body are interconnected. Due to their anatomical proximity to the ear, the nasopharynx, oral and lung microbiomes may, therefore, provide another connection between the gut and ear (Figure 2).

Contrary to what would be expected, due to the close proximity to the ear, studies have found no clear similarities between the adenoid and ear microbiomes (Johnston et al., 2019; Xu J. et al., 2020). However, a number of studies have found similarities between the middle ear microbiome and the nasopharyngeal (Man et al., 2019), oropharyngeal (Lee et al., 2021) or outer ear (Chan et al., 2017) microbiomes in children with otitis media. This research suggests that microbes of the middle ear could originate from these neighboring microbiomes. The nasopharynx and outer ear are also likely sources of pathogens causing infections in the middle ear (Xu et al., 2019; Coleman et al., 2021; Frank et al., 2021). While research has suggested that there is not sufficient evidence of a link between the nasopharyngeal and ear microbiomes (Brugger et al., 2019), other research has indicated that the nasopharynx microbiome may be even more effective for predicting the outcome of otitis media infection than characterizing the middle ear microbiome (Man et al., 2019).

Evidence suggests that the respiratory tract microbiome may also be related to the microbiome of the middle ear (Kolbe et al., 2019; Jörissen et al., 2021). In the microbiome of children with otitis media, respiratory conditions such as asthma influence the ear microbiome composition (Kolbe et al., 2019). Moreover, research has identified that *Streptococcus salivarius* (*S. salivarius*), a bacterial species which populates the mouth and gut in early neonatal development (Kaci et al., 2014) and which is commonly found in the respiratory tract (Jörissen et al., 2021), may have a beneficial influence on the ear microbiome. The K12 strain of *S. salivarius* has specifically been recognized as a potential probiotic for treating otitis media, due to the ability of this strain to target and suppress pathogenic bacteria in the ear (Di Pierro et al., 2015; Chen et al., 2021; Jörissen et al., 2021). The production of bacterial peptides known as ‘bacteriocins’, which can interfere with growth of other bacteria, makes bacterial strains such as *Lactobacillus salivarius* PS7 and *S. salivarius* 24SMB particularly interesting with regard to their potential for treating middle ear infections (Santagati et al., 2012; Marchisio et al., 2015; Cárdenas et al., 2019).

Research also reveals links between the nasopharyngeal microbiome and the brain. A study of severe acute respiratory syndrome coronavirus 2 (SARS-CoV2) showed that the virus can infect the brain and it implicated the mucosal membranes lining the nose as a probable route of entry (Meinhardt et al., 2021). Pathogens that cause meningitis – an infection of the membranes surrounding the brain – were also shown, through a few small case studies, to originate in the nasopharynx (de Andrade et al., 2003; Lauderdale et al., 2005). Several reviews have discussed the role of the nasopharynx and nasopharyngeal microbiome in the development of pneumococcal meningitis (Bogaert et al., 2011; Subramanian et al., 2019; Dietl et al., 2021). Together, these findings suggest that infections originating in the nasopharynx can impact the brain.

Finally, studies of patients with nasopharyngeal carcinoma have found that otitis media infections may be a contributing factor in bacterial infections of the brain (Huang et al., 2003; Fang et al., 2012). Disruptions to the BBB during radiotherapy treatment were suggested to play a role in triggering infections of the brain (Fang et al., 2012). Although these studies do not explicitly state that pathogenic microbes are transferred between the nasopharynx, ear and brain, this may explain these findings.

#### 4.1.3. The labyrinth in motion sickness

Motion sickness provides evidence of yet another connection between the ear, gut and brain (Yates et al., 2014; Figure 2). The vestibule in the labyrinth of the inner ear is required for balance and has been implicated in motion sickness. Patients with lesions in the labyrinth or individuals who undergo surgical removal of the labyrinth are not as prone to motion sickness as healthy individuals (Johnson et al., 1999; Dai et al., 2007). Ultimately, a confusion of signals being received in the brain, from the ears and eyes during motion, are believed to trigger motion sickness (Schmäl, 2013).

Proton channels were shown to play a role in neuronal signaling to the area postrema, within the medulla oblongata, which may contribute to motion sickness in rats (Shinpo et al., 2012). However, there is much debate as to whether the area postrema is in fact involved in motion sickness (Sutton et al., 1988).

Arginine vasopressin, vasopressin receptors and aquaporin make up a signaling pathway within the inner ear which contributes to motion sickness in rats (Xu L.-H. et al., 2020). This research found that rotation (to induce motion sickness) resulted in greater aquaporin 2 expression in structures of the inner ear, as detected using immunohistochemical staining. Additionally, Xu L.-H. et al. (2020) used real time quantitative PCR, Western Blot and enzyme-linked immunosorbent assay (ELISA) techniques to show a sudden, short-lived increase in arginine vasopressin and a more delayed, but longer-lasting, increase in aquaporin in the blood. Xu L.-H. et al. (2020) also found an initial decrease in vasopressin receptor expression in the inner ear. While direct arginine vasopressin treatment and vasopressin receptor agonists promoted motion sickness in animal models, vasopressin receptor antagonists could prevent motion sickness (Xu L.-H. et al., 2020).

While motion sickness largely involves the stomach and can result in disruptions in the movement of and signaling to the gut (Wolf, 1943; Koch, 2014), the microbiome might also be involved in motion sickness. In a small study involving 19 participants, it was found that probiotic treatment may effectively treat motion sickness, particularly seasickness (Srivastava et al., 2021). Moreover Srivastava et al. (2021) carried out additional analysis to explore the functional profiles of the microbiomes of individuals receiving probiotics compared to controls. They found that probiotic treatment resulted in a greater presence of genes encoding enzymes important for breaking down carbohydrates (Srivastava et al., 2021). Finally, endocannabinoid signaling was also implicated in motion sickness, with reductions in systemic endocannabinoid concentrations and receptor numbers observed in individuals experiencing motion sickness (Choukèr et al., 2010).

Although the mechanisms involved remain to be elucidated, there is clear evidence that the ear, brain and gut are all involved in motion sickness. Some of the extracellular mechanisms underlying motion sickness will be discussed further in Section 4.2.

### 4.2. Extracellular mechanisms

**Figure 3:**
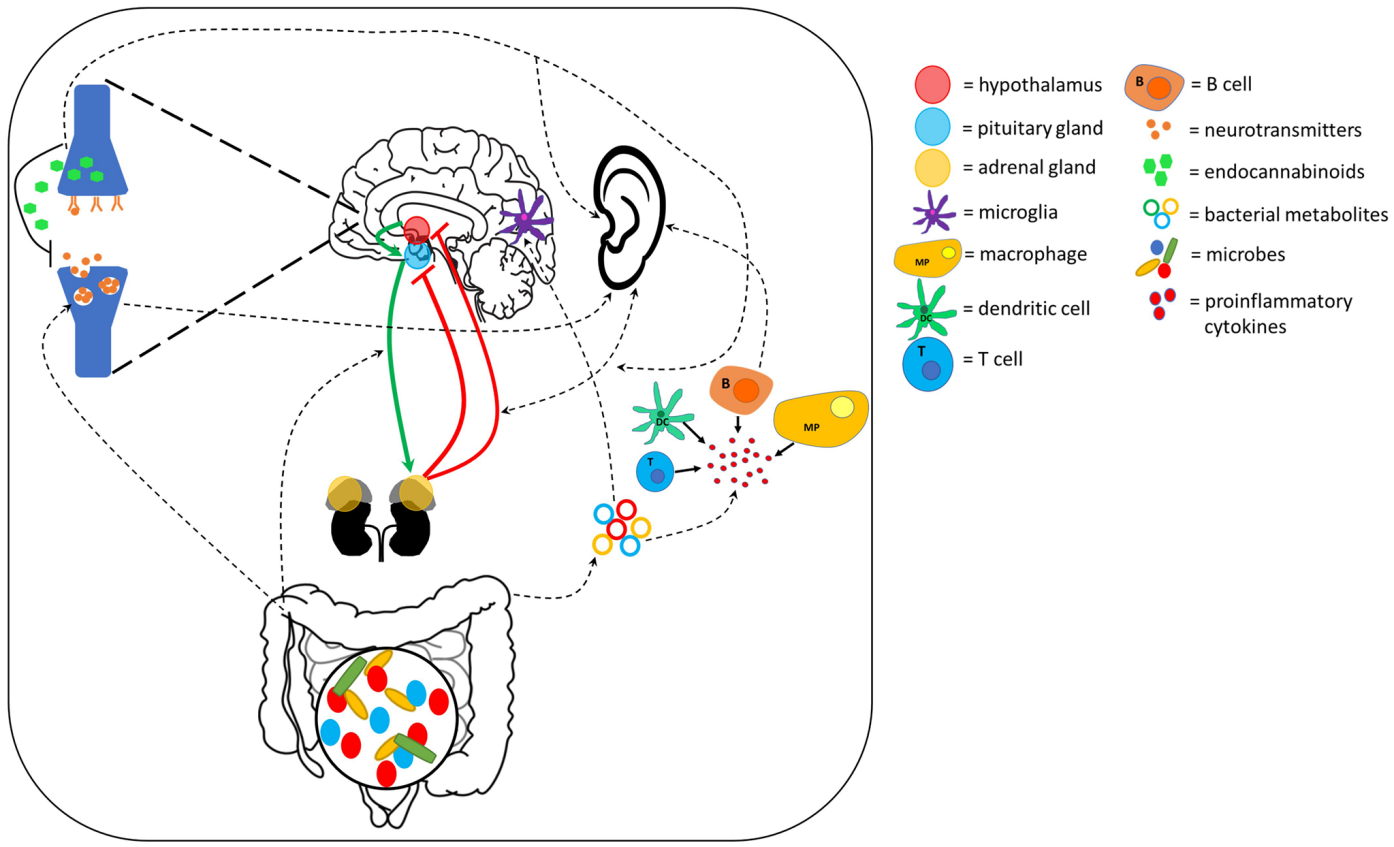
Extracellular signaling pathways connecting the ear, gut and brain. These pathways could also play an important role within the auditory-gut-brain axis. Red and green lines indicate feedback inhibition and the pathway through which hormone release is activated in the HPA axis, respectively. Dotted lines show pathways through which evidence suggests the ear, gut and brain communicate with each other. Components of this figure used the following online resources which all have Creative Commons CC0: https://openclipart.org/detail/38533/brain-side-cutaway by rejon; https://openclipart.org/detail/269019/ear by CCX; https://www.needpix.com/photo/169098/intestines-bowel-guts-intestinal-gastrointestinal-digestive-system-abdominal-biology-science.

There are several extracellular signaling pathways through which gut-brain axis communication can occur. These include HPA axis, neurotransmitter, endocannabinoid and bacterial peptide signaling. Evidence suggests that these may also provide a connection to the auditory system, strengthening the auditory-gut-brain axis hypothesis (Figure 3).

#### 4.2.1. The hypothalamic–pituitary–adrenal axis and stress

The HPA axis, a neuroendocrine signaling mechanism which mediates the release of stress hormones, has been clearly implicated in the gut-brain axis (Sudo et al., 2004; Dinan et al., 2006; Ait-Belgnaoui et al., 2012; Azzam et al., 2017). A greater response to stress is mounted in germ-free mice, as seen by higher corticosterone and adrenocorticotropic hormone (ACTH) levels in limbic regions of the brain compared to specific pathogen-free (SPF) mice (Sudo et al., 2004). Sudo et al. (2004) found that this is exacerbated in mice orally provided with *Escherichia coli* alone. However, in mice inoculated with *Bifidobacterium infantis* alone, or receiving a fecal microbiota transplant from SPF mice, the stress response was restored to SPF mouse levels (Sudo et al., 2004). The authors concluded that during neurodevelopment the composition of the gut microbiome may influence the negative feedback regulation of the HPA axis. Probiotic treatment with *Lactobacillius farciminis* in mice was also found to restore the hypothalamic levels of corticotropin-releasing factor (CRF), which are elevated in response to stress related to restriction of movement (Ait-Belgnaoui et al., 2012).

In patients with irritable bowel syndrome (IBS), circulating ACTH and cortisol levels were significantly elevated, compared to healthy controls, following intravenous CRF treatment (Dinan et al., 2006). This implicates the HPA axis in a gut disorder which can lead to behavioral changes (Gracie et al., 2017), providing evidence of HPA axis function in the human gut-brain axis.

In a small human study, the HPA axis was additionally found to impact the production of ghrelin, a hormone largely produced in the gut (Azzam et al., 2017). Azzam et al. (2017) showed that elevated cortisol levels in the blood (which can be driven by ACTH production), rather than greater ACTH levels in the brain itself, are responsible for inducing ghrelin production. A recent review shows that specific gut microbes and microbial metabolites can regulate ghrelinergic activity (Leeuwendaal et al., 2021). Furthermore, a study in rats showed that peripheral injection of ghrelin into dopaminergic neurons enhances dopamine release, an effect suspected to be due to increased acetylcholine release driving nicotinic cholinergic receptor (nAChR) function (Jerlhag et al., 2012). Interestingly, a review discussing the role of these cholinergic receptors (α-9, α-10nAChR) expressed in the auditory pathway, suggested that these receptors serve as hypothetical drug target sites on which, for example, receptor modulators or antagonists can act to treat hearing loss or complications in the auditory system (Elgoyhen et al., 2009).

Studies have demonstrated the importance of the HPA axis in regulating the activity of the auditory system. Research in mice revealed that pre-exposure to low-level sound can act via the HPA axis to minimize the harmful effects of high decibel sounds in the inner ear following noise-induced trauma (Tahera et al., 2007). Tahera et al. (2007) showed that the HPA axis can be stimulated, leading to a greater release of corticosterone and ACTH into the blood, together with greater expression of glucocorticoid receptors (GRs) in the cochlea and paraventricular nucleus. Ultimately, GRs move to the spiral ganglion neurons where they may impact downstream processes (Tahera et al., 2007). The protection provided by prior auditory stimulation was reversed with GR antagonists or through the surgical removal of the adrenal glands (Tahera et al., 2007).

Murine studies further revealed that corticotropin-releasing factor receptors (CRFR1) are expressed within the inner ear and may act within their own signaling network or interact with the larger HPA axis (Graham and Vetter, 2011). As previously mentioned, probiotics can favorably impact the responses of mice to auditory stressors (Hadizadeh et al., 2019). While mice exposed to auditory stress during fetal development had higher corticosterone, Hadizadeh et al. (2019) showed that prenatal and postnatal oral probiotic treatment can restore corticosterone levels.

In human studies, the HPA axis has been implicated in tinnitus. Adults with tinnitus were found to have slower, less-pronounced cortisol responses following social and psychological stress tests (Hébert and Lupien, 2007). Together these studies indicate that the HPA axis can act on the ear and may, therefore, provide another connection between the auditory system and the gut-brain axis.

#### 4.2.2. Neurotransmitters

Neurotransmitters are chemicals that are released into the synaptic clefts between neurons, in response to an electrical signal, and can drive or inhibit action potentials in adjoining neurons (Patri, 2019). The communication between neurons mediated via neurotransmitters is required for brain functions and behaviors that include learning and memory, sleep and mood (Mendelson, 2001; Seyedabadi et al., 2014; Wilkinson and Sanacora, 2019). Although neurotransmitters are generally produced within the nerves themselves (Oda, 1999; Tian et al., 1999; Patri, 2019), immune cells such as T cells may also be a source of neurotransmitters (Rosas-Ballina et al., 2011).

Additionally, bacteria in the gut have been found to release neurotransmitters as a product of their metabolic activity (Barrett et al., 2012). Probiotic treatment in mice has been shown to influence the levels of neurotransmitters, such as gamma-aminobutyric acid (GABA) and glutamate, in the brain (Janik et al., 2016). Providing animals with microbial transplantations to mimic autism- and schizophrenia-like profiles resulted in changes, respectively, in the behavior of their young (Qi et al., 2021) or in the animals themselves (Zhu et al., 2020). Moreover, neurotransmitter signaling was found to be affected in these studies, albeit temporarily in the schizophrenia models (Zhu et al., 2020; Qi et al., 2021). While the offspring of dams provided with microbes from patients with autism displayed lower systemic GABA and norepinephrine (Qi et al., 2021), animal models of schizophrenia had reduced systemic dopamine and lower enteric GABA (Zhu et al., 2020).

These chemicals, which play a crucial role in signaling between the brain and many parts of the body (including the gut), provide a further mechanism which may connect the gut-brain axis to the ear. Animal studies have shown the importance of GABA, an inhibitory neurotransmitter, in the long-term survival and functionality of inner ear nerves (Maison et al., 2006). Further, a relationship was observed between age-related hearing loss and a reduction in various neurotransmitter receptors (acetylcholine, N-methyl-D-aspartate and GABA receptors) on spiral ganglia of the inner ear in mice (Tang et al., 2014). This would limit the ability of neurotransmitters to act in the ear. A study in rats also found that acetylcholine-specific (“cholinergic”) neurons may contribute to motion sickness, as introducing acetylcholine receptor inhibitors was able to reduce motion sickness-related symptoms (Qi et al., 2019). This finding supports a potential contribution to auditory-gut-brain axis signaling.

Furthermore, research in humans revealed a contribution of neurotransmitters in tinnitus (Sedley et al., 2015). Sedley et al. (2015) used magnetic resonance spectroscopy to investigate metabolic activity and GABA levels in the brain. They found that GABA levels were lower in the auditory cortices of adults presenting with unilateral tinnitus compared to healthy controls of a similar age and with comparable hearing test outcomes (Sedley et al., 2015).

Therefore, neurotransmitters play important roles in both the inner ear (Qi et al., 2019) and primary auditory cortex (Sedley et al., 2015).

#### 4.2.3. Endocannabinoids

Endocannabinoids are interesting in that they prevent the release of neurotransmitters from the presynaptic cleft in a feedback manner, where they are themselves released from the postsynaptic neurons (Alger, 2013). They play a role in the gut-brain axis, acting on signaling pathways such as the vagus nerve and HPA axis (DiPatrizio, 2016; Sharkey and Wiley, 2016). In a study where the Cre/loxP system was used to knock out cannabinoid receptors specifically in neurons of the vagus nerve, motility of the gut was increased in mice (Vianna et al., 2012). Further, restraint stress activates the HPA axis and results in the release of glucocorticoids in mice – a process that endocannabinoids inhibit once the stress-triggering factor has been removed (Hill et al., 2011). Hill et al. (2011) found that knocking out the cannabinoid receptors, or treating mice with cannabinoid receptor antagonists, resulted in corticosterone being released at higher levels for a longer period of time – indicating an extended stress response in the absence of endocannabinoid signaling.

Endocannabinoid/cannabinoid receptor signaling was found to influence the negative feedback loops of the HPA axis in rats following noise-induced stress – revealing a connection to the auditory system (Newsom et al., 2020). Newsom et al. (2020) showed that auditory-related stress led to higher systemic levels of stress hormones, increased transcription of genes encoding cannabinoid receptors in the adrenal gland and greater transcription of the c-fos gene within various brain regions including central auditory regions. Furthermore, antagonists to cannabinoid receptors drove elevations in blood corticosterone in these mice (Newsom et al., 2020). This suggests yet another possible signaling pathway in the auditory-gut-brain axis.

Activation of cannabinoid receptors was also found to safeguard against inflammation and oxidative stress in the inner ears of mice, following chemotherapeutic treatment which can lead to hearing loss (Ghosh et al., 2018). Among the targets of cannabinoid receptor signaling identified by Ghosh et al. (2018) were sodium/potassium pumps on the stria vascularis, which keep potassium levels high in the endolymph (Wangemann, 2006). A study in mice has shown that interfering with the function of these transporters, resulting in a disruption to endolymph homeostasis, can impact the development of the cochlea and affect hearing (Li et al., 2013). Therefore, endocannabinoid signaling via cannabinoid receptors in the ear can maintain homeostasis in the ear and ultimately prevent damage to the inner ear. Additionally, through animal research, endocannabinoid or cannabinoid signaling via cannabinoid 2 receptors has been shown to influence the differentiation of microglia – immune cells localized to the central nervous system (CNS) (Delage et al., 2021; Paolicelli et al., 2022); in this case they shift from a pro- to anti-inflammatory state, and pro-inflammatory cytokine release is reduced (Tanaka et al., 2020).

The expression of cannabinoid receptors in the neurons of the central auditory system differs from that of many other neurons, and this is important for controlling neurotransmitter signaling through these neurons (Zhao et al., 2009). In particular, Zhao et al. (2009) found that cannabinoid receptors are sparser on inhibitory synapses and are expressed in greater numbers on excitatory synapses of the auditory brainstem. Ultimately, endocannabinoids are less able to restrict inhibitory signaling in auditory neurons – as there are fewer receptors to act on in the inhibitory sources – but they can still inhibit sources of excitatory signaling (Zhao et al., 2009). In the central auditory system of rats, cannabinoid receptors were also implicated in the phenomenon of “stimulus specific adaptation,” by which auditory stimuli of a frequency of sound commonly experienced by the auditory system, drive a less substantial response in the brain (Valdés-Baizabal et al., 2017).

Finally, as previously discussed, motion sickness provides another mechanism by which the auditory system, the gut and brain may be connected. A link between endocannabinoid signaling and motion sickness was established in individuals who experience zero-gravity during flights (Choukèr et al., 2010). In this study by Choukèr et al. (2010), the expression of cannabinoid receptors on white blood cells and the blood concentration of endocannabinoids were shown to be reduced in individuals with motion sickness. Moreover, a connection between endocannabinoid signaling and the HPA axis was also shown in this study, within the context of motion sickness (Choukèr et al., 2010).

#### 4.2.4. Bacterial metabolites

Bacteria in the gut digest food and generate metabolites which are essential for the human body. Metabolites such as sodium butyrate and acetate are involved in gut-brain axis signaling and may play a role in various neurological disorders such as Alzheimer’s disease (Govindarajan et al., 2011), Parkinson’s disease (Liu et al., 2017) and MS (Olsson et al., 2021), as seen in animal studies.

Additionally, bacterial metabolites can impact the immune system by triggering the differentiation of regulatory T (Treg) cells (Arpaia et al., 2013; Smith et al., 2013). Mice lacking gut microbes have a severe reduction in Treg cells, while SCFA treatment can renew the ability of Treg cell differentiation (Smith et al., 2013). Thus, SCFAs can affect the regulation of inflammation via Treg cells.

In the brain, bacterial metabolites such as SCFAs have been shown to regulate microglial development and activity in mice (Erny et al., 2015). By knocking out SCFA receptors, Erny et al. (2015) showed that mice displayed abnormal microglial development. Repeated administration of broad-spectrum antibiotics (metronidazole, cefoxitin, and gentamicin) to male and female mice for a month, caused a reduction in gut bacteria leading to under-development of microglia (Erny et al., 2015). Furthermore, antibiotic treatment has been found to affect microglial gene expression in a different way for male versus female mice, and at different stages of development (Thion et al., 2018). Collectively, these studies highlight the importance of the microbiome in shaping the CNS immune system.

The effects of bacterial metabolites on the immune system can extend to the ear. Mouse models of conditions such as atopic dermatitis can assist in better understanding the role of the immune system in the ear and how to influence its activity. As will be discussed, probiotic treatment in mice can alter the immune response to atopic dermatitis of the ear (Fang et al., 2020). Furthermore, Fang et al. (2020) found that elevations in butyric acid shifted the immune response away from a type 2 response that is characteristic of atopic dermatitis, to a type 1 immune response − favoring better disease outcomes. Therefore, metabolic activity of gut microbes can impact the immune response in the ear. A recent study showed that the ingestion or direct application of polysaccharides derived from the *Tremella fuciformis* fungus on the affected ears of mice, could alter microbial and metabolic profiles of the gut and ultimately provide relief from atopic dermatitis symptoms (Xie et al., 2022).

The G protein-coupled receptor (GPCR) GPR43 is an SCFA receptor which has also been implicated in mouse models of atopic dermatitis (Kang and Im, 2020) and psoriasis (Nadeem et al., 2017), an inflammatory disease of the skin. While Kang and Im (2020) found that a GPR43 receptor agonist could dampen the type 2 response and minimize atopic dermatitis symptoms, Nadeem et al. (2017) found that agonists for the receptor escalated the levels of skin inflammation on the ears of mice with psoriasis. These studies together highlight the role of SCFA receptors in allergic and inflammatory responses of the immune system within the ear.

Sodium butyrate is an SCFA which can act via GPCRs to inhibit histone deacetylase (HDAC), leading to reduced neuronal death and oxidative stress (Zhou et al., 2021). In addition to the protective role sodium butyrate plays in the nervous system, the inhibition of HDAC by sodium butyrate was also shown to improve hearing in guinea pigs previously impacted by the antibiotic gentamycin (Wang et al., 2015b). This indicates that SCFAs can impact hearing at an epigenetic level.

Finally, research has found that SCFAs can act via the vagus nerve (Onyszkiewicz et al., 2019). Although this study makes no mention of the ear, the action of SCFAs on the vagus nerve – which provides an anatomical connection between the ear, gut and brain – may provide another avenue by which the gut microbes can interact with the auditory system. However, this would need to be investigated further.

### 4.3. Immune system mechanisms

**Figure 4:**
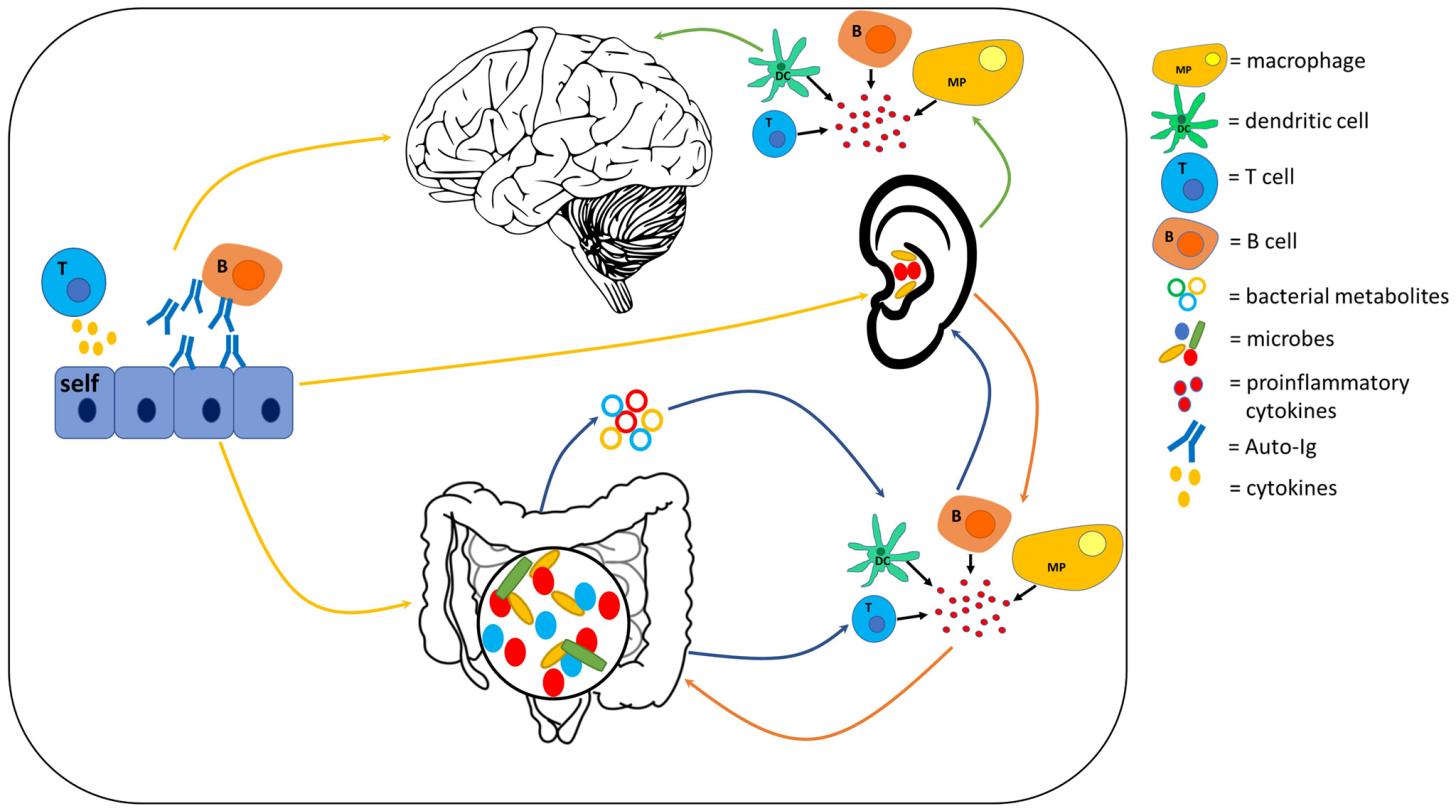
Potential inflammatory and autoimmune mechanisms which could provide communication pathways within the auditory-gut-brain axis. Gut-to-auditory and auditory-to-gut signaling pathways involving inflammation are shown as blue and orange arrows respectively; inflammatory pathways of the auditory-brain axis are shown as green arrows; yellow arrows represent the contribution of autoimmunity in the auditory-gut-brain axis. Components of this figure used the following online resources which all have Creative Commons CC0: https://openclipart.org/detail/35791/brain-01 by rejon; https://openclipart.org/detail/269019/ear by CCX; https://www.needpix.com/photo/169098/intestines-bowel-guts-intestinal-gastrointestinal-digestive-system-abdominal-biology-science.

The immune system plays a vital defence role in the body and represents another mechanism which connects gut microbes to the brain. The gut is a major hub for the immune system and trains immune cells to distinguish between pathogens, beneficial bacteria (commensals) and cells belonging to the host (Zheng et al., 2020). Gut bacteria may also contribute to inflammatory responses in the brain by influencing cytokine signaling (Lin et al., 2019). The potential role of the immune system in connecting the ear, gut and brain (Figure 4) will be discussed under two sub-headings: inflammation and autoimmune disease.

#### 4.3.1. Inflammation

Several studies have demonstrated the effects of the microbiome on the immune response in the ear by artificially inducing disease symptoms in animal ear models. A 2011 mouse study looked at the contribution of *Candida albicans* (*C. albicans*) – a microbial species present in the human gut but not naturally occurring in mice – in various inflammatory diseases (Sonoyama et al., 2011). By applying 2,4-dinitrofluorobenzene on the ear, Sonoyama et al. (2011) mimicked contact hypersensitivity. The authors were able to show that when introducing *C. albicans* into the guts of mice, a greater degree of ear swelling occurred accompanied by higher levels of proinflammatory cytokines (IL-1β, IL-6 and TNF-α) in the ear (Sonoyama et al., 2011). These bacteria also resulted in worse outcomes for other inflammatory disease models in mice, such as rheumatoid arthritis and allergic diarrhea (Sonoyama et al., 2011).

Another study in mice investigated whether Urolithin A (UA), a microbial metabolite generated from plant-based ellagic acid, could regulate the neutrophil myeloperoxidase defence mechanism – an oxidative stress catalyst which leads to inflammation and tissue damage resulting from cell death (Saha et al., 2016). To investigate the potential of UA, Saha et al. (2016) used phorbol myristate acetate (PMA) to induce edema and model neutrophil-related oxidative stress in the ears of mice (Wu et al., 2020). An oral supply of UA was shown to counteract myeloperoxidase activity and inflammation within the ear (Saha et al., 2016). This finding indicates that microbial metabolites may impact the immune system and oxidative stress response following immune activation in the ear.

Various outer, middle and inner ear disorders in humans may be associated with inflammation of the gut (Fousekis et al., 2018). In children who have experienced otitis media infections in the middle ear, the risk of inflammatory bowel syndrome is greater (Shaw et al., 2013). Shaw et al. (2013) postulated that this may be due to antibiotic treatment, rather than the infection itself. SNHL is more prevalent in individuals with ulcerative colitis and Crohn’s disease compared to control participants – although the degree of severity observed in this study was mild (Akbayir et al., 2005). Importantly, this suggests that hearing loss related to inflammation in the gut can be subtle and may often remain undiagnosed.

Hearing loss was also linked to inflammation in the brain, notably by recruitment of microglia (Wang et al., 2019), which play important roles in health and disease (Delage et al., 2021; Paolicelli et al., 2022). Following tinnitus and hearing loss driven by noise exposure, greater microglial reactivity in terms of morphological changes and inflammatory cytokine levels (such as TNF-α and IL-1β) were observed in the central auditory system of mice (Wang et al., 2019). In mice for which the gene for proinflammatory cytokine TNF-α was genetically removed, Wang et al. (2019) found a reduction of tinnitus, accompanied by dampened inflammation in the auditory cortex and restored auditory brainstem responses, reaching similar levels to wild-type control mice. Further, treatment with 3,6′-dithiothalidomide (TNF-α inhibitor), to prevent microglial reactivity, also led to reduced tinnitus (Wang et al., 2019).

#### 4.3.2. Autoimmunity

Autoimmune diseases occur as a result of the host immune system being unable to discern host cells from pathogens, leading to host cells being attacked (Lleo et al., 2010). A number of autoimmune diseases can lead to hearing loss (Gawron et al., 2004; Hellmann et al., 2011; Berker et al., 2012; Solmaz et al., 2012; Mijovic et al., 2013). Of these, diseases such as MS and celiac disease were shown to impact the gut and brain, suggesting another aspect in which the immune system could modulate the auditory-gut-brain axis.

##### 4.3.2.1. Multiple sclerosis

MS is an autoimmune disease in which damage occurs to the myelin sheath that coats neurons, leading to CNS alteration and neurological dysfunction (Kern et al., 2009; Stadelmann et al., 2011). Although it is not clear whether MS is a typical autoimmune disease whereby auto-antibodies drive damage, it is agreed that the host immune system is still responsible for this disease (Lemus et al., 2018; Prineas and Parratt, 2018; Höftberger et al., 2022). Previous research has shown that, in addition to the gut microbiome being disrupted in patients with MS, the ability of Treg cells to differentiate is impacted in germ free mice given a fecal microbiota transfer from MS patients (Cekanaviciute et al., 2017). Treg cells play an important role in regulating the immune system, thus disrupting their function can lead to the immune system going unchecked and attacking the host. A study in mice also showed that the ENS can be targeted by the immune system in MS, suggesting that this may also contribute to driving gastrointestinal complications in MS patients (Spear et al., 2018). A review of animal and human research has shown the success of probiotic treatment for MS, in terms of improving clinical outcomes (Jiang et al., 2021). Furthermore, a meta-analysis carried out by Jiang et al. (2021) using data from three studies found that probiotics can improve different psychological health measures in individuals with MS.

In addition to affecting the brain and gut, hearing loss was found to occur in MS patients, albeit in a relatively small proportion, and may in fact be an early symptom of MS (Marangos, 1996; Hellmann et al., 2011). SNHL often occurs suddenly, yet is temporary in nature (Hellmann et al., 2011). Research indicates that the cause of SNHL in MS may be damage to the cochlear nerve (Marangos, 1996). This might be expected to lead to downstream effects on the central auditory system, however further research would be required to investigate this involvement. Additionally, vertigo has been reported in individuals with MS and may indicate an effect of MS on the vestibular system (Rae-Grant et al., 1999; Alpini et al., 2001).

##### 4.3.2.2. Celiac disease

Celiac disease (CD) is a disorder of the gut, characterized by an inflammatory response following ingestion of gluten (Koning et al., 2005; Meresse et al., 2009). The composition of the gut microbiome is altered in patients with CD (De Palma et al., 2010), with a greater presence of species from the Staphylococcus genus observed (Sánchez et al., 2012). Immunoglobulin-A (IgA), the main antibody in the gut, plays several important roles including neutralizing pathogens (Corthésy, 2013; Gutzeit et al., 2014; de Sousa-Pereira and Woof, 2019). The proportion of IgA-neutralized bacteria in the gut is lower in CD patients (De Palma et al., 2010). Collectively, a greater presence of pathogenic bacteria – together with the reduced ability of the gut immune system to provide a protective IgA response to neutralize these species – may trigger inflammation and increase bacterial leakage.

A number of studies indicate that probiotics could be used to treat CD (De Angelis et al., 2006; Quagliariello et al., 2016; Francavilla et al., 2019). However, research also found that probiotics are not effective in infants who are genetically predisposed to CD and may make them more prone to an autoimmune response (Uusitalo et al., 2019). Previous studies have additionally found that CD may impact the brain, particularly in white matter regions (Kieslich et al., 2001; Croall et al., 2020).

In addition to affecting the gut and brain, SNHL has been observed in patients with CD (Solmaz et al., 2012). Although some studies show that hearing loss is more frequently observed in adults (Leggio et al., 2007) and children (Hizli et al., 2011) with CD, other research suggests that patients with CD are at no greater risk for hearing loss than healthy controls (Bükülmez et al., 2013; Urganci et al., 2015).

While the co-occurrence of gut, brain and hearing disorders in these autoimmune diseases indicates that autoimmunity may be a mechanism involved in the auditory-gut-brain axis, more research is warranted to determine whether these co-morbidities are functionally related or only represent an independent association.

### 4.4. Dietary mechanisms

**Figure 5:**
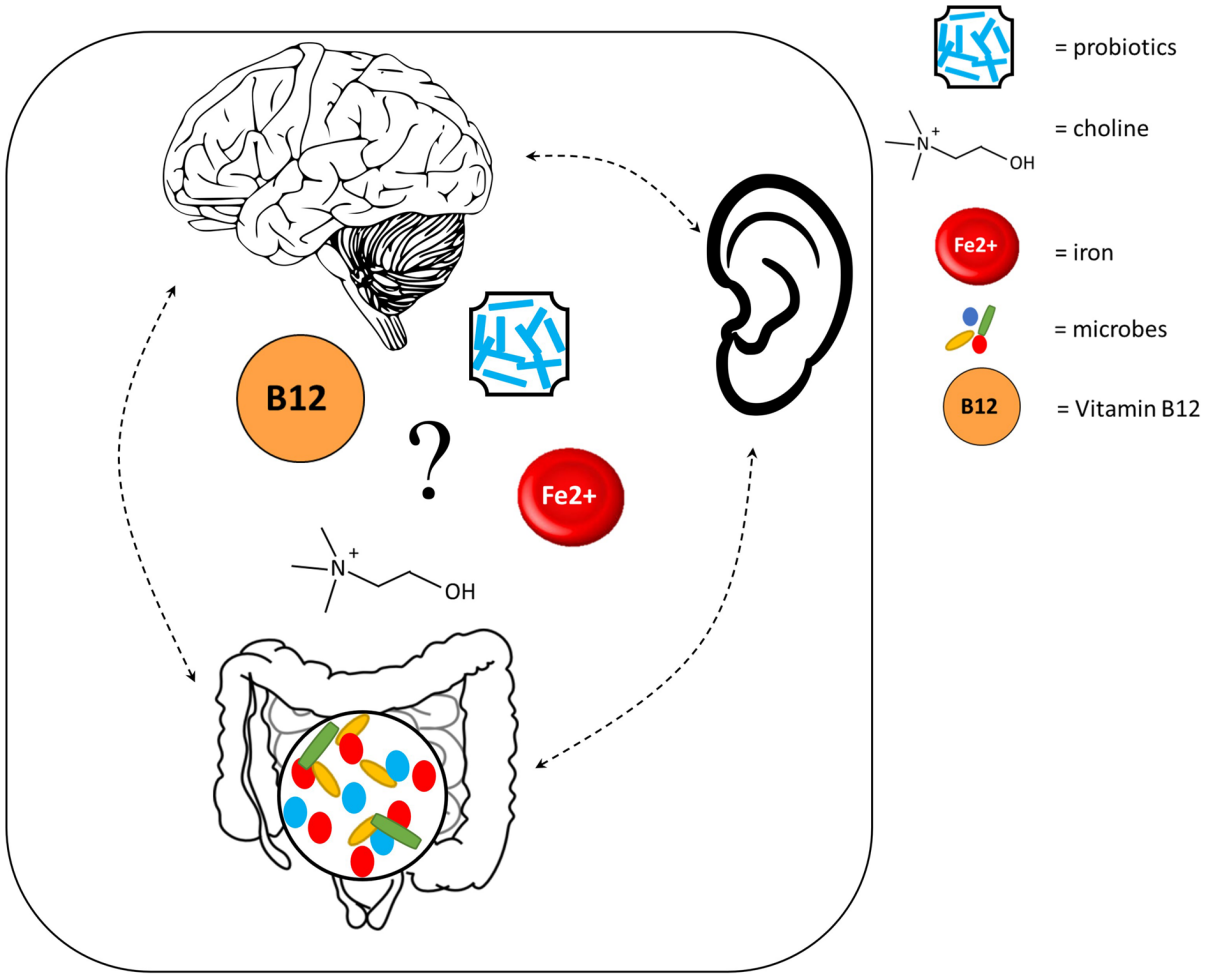
Dietary factors which may play a key role in the auditory-gut-brain axis. Components of this figure used the following online resources which all have Creative Commons CC0: https://openclipart.org/detail/35791/brain-01 by rejon; https://openclipart.org/detail/269019/ear by CCX; https://www.needpix.com/photo/169098/intestines-bowel-guts-intestinal-gastrointestinal-digestive-system-abdominal-biology-science.

Other mechanisms through which the auditory system may be connected to the gut and brain comprise diets and supplements. Deficiencies in several dietary factors, such as iodine, zinc and omega-3 polyunsaturated fatty acids (PUFA), can impact neurodevelopment (Lange and Lange, 2021). Further, iodine and omega-3 PUFA deficiencies are associated with hearing loss (Gopinath et al., 2010; Melse-Boonstra and Mackenzie, 2013), while zinc supplementation shows promise for treating SNHL (Yang et al., 2011), suggesting that these nutrients also impact the auditory system. However, the causal roles that nutrient deficiencies play in hearing loss remain to be understood. Here we focus on four additional dietary factors that may represent potential key players in the auditory-gut-brain axis (Figure 5).

#### 4.4.1. Probiotics

Probiotics have been used to treat both gut (Marteau et al., 2002; Guarino et al., 2015; Jia et al., 2018) and neurological (Akbari et al., 2016; Dutta et al., 2019) disorders in patients. Probiotics can play an important role in re-establishing the relative abundance of microbes and minimizing gastrointestinal symptoms resulting from dysbiosis associated with prevalent pathogenic species in the gut (Hickson et al., 2007; Gao et al., 2010). More specifically, Hickson et al. (2007) showed that a mixture of *Lactobacillus* species (*L. casei and L. bulgaricus*) and *Streptococcus thermophilus* was an effective treatment strategy for individuals receiving courses of antibiotics, to minimize cases of *Clostridium* infections and resulting diarrhea. A mechanism by which probiotics can outcompete *Clostridium difficile* and reduce the toxic effects of this pathogen involves altering the environmental pH (Wei et al., 2018).

Additionally, probiotics may reduce the permeability of the gut and minimize leakage of LPS into the blood following acute psychological stress in rats (Ait-Belgnaoui et al., 2012). Ait-Belgnaoui et al. (2012) also found that probiotics can impact the stress response via the HPA axis (as was discussed in Section 4.2.1). Overall, these findings indicate that probiotics influence the gut-brain axis.

Probiotics may also have an effect on the ear and ear infections can be treated by the ingestion of probiotics. In otitis media infections, for example, the oral intake of probiotics was identified as an effective treatment (Rautava et al., 2008; Di Pierro et al., 2016). Probiotics have further been shown to influence the immune response to allergic insults in the ears of mice, leading to a reduction of type 2 IgE response and fewer mast cells being recruited to the ear (Fang et al., 2020). Additionally, Fang et al. (2020) found that the microbiome communities and function, specifically SCFA production, within the gut was altered with the introduction of *Bifidobacterium adolescentis*. In particular, an increase in propionic and butyric acids was noted in mice with atopic dermatitis following treatment with the FJSYC5M10 strain, compared to untreated mice with atopic dermatitis (Fang et al., 2020). Finally, as previously discussed in Section 3, probiotics can also have an impact on hearing at the central auditory system level (Oike et al., 2016; Figure 5).

When considering probiotics for therapeutic purposes, it is important to consider that it does not come without risk. In a review of relevant literature, Sotoudegan et al. (2019) identified various possible negative outcomes of probiotic treatment, for which immunocompromised individuals, the very young and elderly were found to be the most vulnerable. These included the interchange of genetic material between probiotics and commensals, overexcitation of the immune system and bacterial invasion of the bloodstream (Sotoudegan et al., 2019).

#### 4.4.2. Choline

As the human body is unable to generate sufficient choline for its metabolic needs, dietary intake is required (Zeisel and Da Costa, 2009). Research in animals (Ye et al., 2018) and humans (Spencer et al., 2011) suggests that choline deficiencies can shift the composition of the gut microbiome. It should be noted, however, that in the study by Ye et al. (2018) the diet provided to mice also lacked methionine.

Choline supplementation in pregnant mothers has beneficial effects on brain development, leading to improved neurocognitive outcomes in infants (Wu et al., 2012; Caudill et al., 2018; Jacobson et al., 2018). For example, providing mothers with choline supplements during pregnancy was shown to reduce the neuroanatomical injury caused to infants by prenatal alcohol exposure (Warton et al., 2020). Choline is an essential precursor in the production of acetylcholine, a major neurotransmitter for neurocognitive function (Oda, 1999). Depleted choline or acetylcholine, or disruptions to choline-dependent signaling, have been implicated in various inflammatory conditions such as Alzheimer’s disease (Spencer et al., 2011; Hung and Fu, 2017; Hampel et al., 2018; Al-Humadi et al., 2019). Previous research suggests that acetylcholine may act via the vagus nerve to reduce inflammation, thus providing a modulatory link with the immune system which is also referred to as the “cholinergic anti-inflammatory pathway” (Borovikova et al., 2000). Indeed, research in humans has also shown that greater levels of choline during pregnancy can counteract the harmful effects that maternal infections can have on infant neurodevelopment (Freedman et al., 2019). In particular, Freedman et al. (2019) assessed the ability of infants to filter out unnecessary auditory stimuli while sleeping, as measured by auditory evoked potentials, showing better outcomes in infants born to mothers with higher choline levels.

Animal studies have shown the value of choline supplementation in the hearing of offspring (Cheng et al., 2008; Stevens et al., 2008) and in some neuropsychological disorders (Cano et al., 2021). Choline is able to restore the ability to inhibit auditory stimuli, a common measure of sensorimotor gating in mouse models of schizophrenia following an initial auditory priming stimulus (Stevens et al., 2008). Further, disrupted cholinergic signaling in the amygdala was implicated in prepulse inhibition (PPI) – a measure of the subconscious attenuation of response to signals in the acoustic startle (Cano et al., 2021). Previous studies also showed that brain inflammation, as seen in schizophrenia, can be treated with alpha-7 nAChR (α-7nAChR) agonists or by stimulating the vagus nerve (Corsi-Zuelli et al., 2017). This pathway further reinforces the relationship of the gut-brain axis, in auditory disorders, impaired sensorimotor gating and pathogenesis of neuropsychiatric diseases like schizophrenia. Moreover, stimulation of the vagus nerve was found to effectively treat the symptoms of schizophrenia by preventing excessive signaling from the hippocampus, which disrupts dopamine signaling (Perez et al., 2014). Additionally, a study in guinea pigs found that the choline transporter CTL-2 may be involved in autoimmunity-related hearing loss – although it is unclear whether this transporter does in fact transport choline (Nair et al., 2004).

Choline supplements particularly benefit the central auditory system in individuals requiring hearing aids due to hearing deterioration with age (Na et al., 2021). Na et al. (2021) provided a choline alfoscerate supplement, which can be converted into acetylcholine, and this was shown to have a neuroprotective role using animal epilepsy models (Lee et al., 2017). Therefore, choline supplements appear to assist with hearing mainly at the central auditory system level.

As with probiotics, choline supplementation has been linked to possible negative health outcomes. Bacterial enzymes in the gut digest choline into trimethylamine, which is then transported to the liver where it undergoes an oxidation step to form trimethylamine N-oxide (TMAO) (Vogt et al., 2018). Increased TMAO levels have been implicated in blockage of blood vessels leading to vascular senescence, atherosclerotic heart disease (Wang et al., 2011) and inflammatory bowel disease (Santoru et al., 2017).

#### 4.4.3. Iron

Iron plays an important role in the nervous system. Iron deficiencies can impact brain development in infants and children both in terms of cognitive and motor skill development (Walter et al., 1989; Shafir et al., 2008; Fuglestad et al., 2016). Animal research has shown that a lack of iron, particularly during critical developmental stages, can interfere with axonal myelination, disrupt neurotransmitter activity and alter metabolite concentrations in the hippocampus – including increased GABA, N-acetyl aspartate and glutamate (Kwik-Uribe et al., 2000; Beard et al., 2003; Rao et al., 2003; Wu et al., 2008). Furthermore, inflammatory bowel disease has been associated with iron deficiency anemia in humans (Semrin et al., 2006; Martinelli et al., 2016). Therefore, a lack of iron can affect both the brain and the gut.

Hepcidin – a hormone which acts on iron transporters to inhibit the uptake of iron in the gut – plays an important role in controlling iron levels in the brain and was shown to be influenced by inflammation (Vela, 2018). Research has found that circulating levels of C-reactive protein (a marker of inflammation) are linked to higher levels of hepcidin, resulting in reduced iron resorption (Semrin et al., 2006, Martinelli et al., 2016). Greater systemic levels of hepcidin and C-reactive protein have further been found to affect hearing, and these proteins are additionally associated with the IL-6 response (Al-Katib et al., 2018).

In addition to affecting the gut and brain, iron deficiency anemia has been found to cause SNHL in both animals (Jougleux et al., 2011) and humans (Schieffer et al., 2017b). This indicates that a lack of iron may specifically impact activity of the cochlea or inner ear. In adults a combination of SNHL and conductive hearing loss may occur with iron deficiency, which the authors postulated may be linked to the supply of blood and oxygen to the inner ear (Schieffer et al., 2017a).

Fetal development is a crucial time in the establishment of the auditory system. Research in guinea pigs has shown that an iron-deficient diet during pregnancy results in damage to the hair cells of the cochlea in offspring (Yu et al., 2014). More specifically, Yu et al. (2014) found that this damage occurs as a result of caspase-driven apoptosis. Although other animal research has shown the role of caspase-driven apoptosis in causing hearing loss (Han et al., 2015), caspase has also been identified as an important factor in development of the inner ear (particularly the vestibule) and transmission of auditory signals (Makishima et al., 2011). Mice lacking caspase 3 were found by Makishima et al. (2011) to have much higher auditory brainstem response thresholds indicative of hearing loss.

Iron deficiency anemia has been shown to impact development of the central auditory system in infants, which could have long-term implications for their auditory function (Sundagumaran and Seethapathy, 2019). A recent study in infants ruled out the possibility that a lack of iron interferes upstream with the amplification role of outer hair cells in the cochlea (Sundagumaran and Seethapathy, 2020). Infants exposed to lower levels of iron *in utero*, as determined from umbilical cord blood tests, display delayed signal conduction in their auditory brainstem response measures (Amin et al., 2013). Amin et al. (2013) suggest that this is indicative of disruptions to nerve myelination in the central auditory system.

Finally, acute otitis media infections of the middle ear are more commonly observed in children with iron deficiency anemia (Golz et al., 2001), while treating iron deficiency anemia has been found to prevent the recurrence of otitis media infections (Venugopal et al., 2018). Moreover, iron deficiency has been found to cause inflammation in the middle ear of children, which the authors speculate may lead to oxidative stress (Purwanto et al., 2021).

Optimal iron intake is clearly essential in the auditory system, gut and brain, but tissue levels need to be tightly regulated. When taking supplements, a surplus of iron must be avoided as unused iron may accelerate the production of free radicals. This can lead to tissue damage, particularly in the heart and liver, and can result in inflammation, cancerous growths or ultimately result in failure of these organs (Attar, 2020).

#### 4.4.4. Vitamin B12

The influence of vitamin B12 on the gut microbiome has been reviewed in depth (Uebanso et al., 2020; Guetterman et al., 2022). While some microbes of the gut are capable of producing vitamin B12 (Martens et al., 2002; Santos et al., 2008; Magnúsdóttir et al., 2015), and the potential for probiotics containing vitamin B12-producing microbes has been demonstrated in zebrafish (Qi et al., 2023), other bacteria are reliant on receiving vitamin B12 that can be metabolized and used particularly for enzymatic activity (Rodionov et al., 2003; Degnan et al., 2014a,b). *In vitro* studies found that vitamin B12 supplements could alter the balance of bacterial species in samples collected from patients with vitamin B12 deficiencies (Xu et al., 2018). Interestingly, a study found that vitamin B12 supplements had little effect on the microbiome of C57Bl/6 mice with a healthy gut, but higher vitamin B12 levels led to greater damage specifically in mice modeling colitis (Lurz et al., 2020). The importance of distinguishing between the effects of various forms of vitamin B12 on the gut microbiome has, however, been highlighted in both animal and human studies (Xu et al., 2018; Zhu et al., 2018). Zhu et al. (2018) found that when treating gut disorders such as IBD, some forms of vitamin B12 may exacerbate symptoms by favoring bacterial species such as *E. coli*.

Inadequate vitamin B12 levels have been implicated in various neurological disorders including Alzheimer’s, schizophrenia and dementia, and an inverse correlation can be seen between age and vitamin B12 concentrations in the brain (Wang et al., 2001; Zhang et al., 2016; Jatoi et al., 2020; Shrestha et al., 2022). Lower levels of methyl cobalamin, a specific form of vitamin B12, have been found to contribute to reduced antioxidant compounds and methylation of DNA in individuals with schizophrenia and autism, which could, respectively, lead to greater oxidative stress and interfere with DNA transcription (Zhang et al., 2016). Animal studies have found that vitamin B12 may participate in neuronal recovery following damage (Okada et al., 2010; Sun et al., 2012; Wu et al., 2019), which may be due to a role in BDNF production (Sun et al., 2012) or in methylation processes (Okada et al., 2010). Moreover, sufficient *in-utero* vitamin B12 levels are crucial for neurodevelopment and long-term cognitive performance in infants and children (Bhate et al., 2012; Lai et al., 2019; Golding et al., 2021). Vitamin B12 may also be implicated in the gut-brain axis, as functional analysis has found that the microbiome of children receiving treatment for ADHD has fewer genes required for vitamin B12 production, compared to untreated children (Stiernborg et al., 2023).

In addition to the role of vitamin B12 in the gut and brain, studies have found that insufficient levels may be implicated in hearing loss (Houston et al., 1999; Péneau et al., 2013) and tinnitus (Lasisi et al., 2012; Singh et al., 2016; Kisli and Saçmacı, 2019). In individuals with vitamin B12 deficiency-related tinnitus, the impacts can be detected at a central auditory level, as measured by auditory brainstem response (Kisli and Saçmacı, 2019). Mechanisms have been suggested for how vitamin B12 deficiencies might impact the auditory system, including disruption of blood supply to the inner ear or interference in cochlear nerve myelination (Houston et al., 1999; Kisli and Saçmacı, 2019). Vitamin B12 supplements have also been shown to alleviate tinnitus-related symptoms (Singh et al., 2016) and to improve hearing outcomes, specifically in older women with vitamin B12 deficiencies (Houston et al., 1999, Péneau et al., 2013). As such, this dietary factor could provide a link between the gut-brain axis and auditory system.

## 5. Perspective and future work

**Figure 6:**
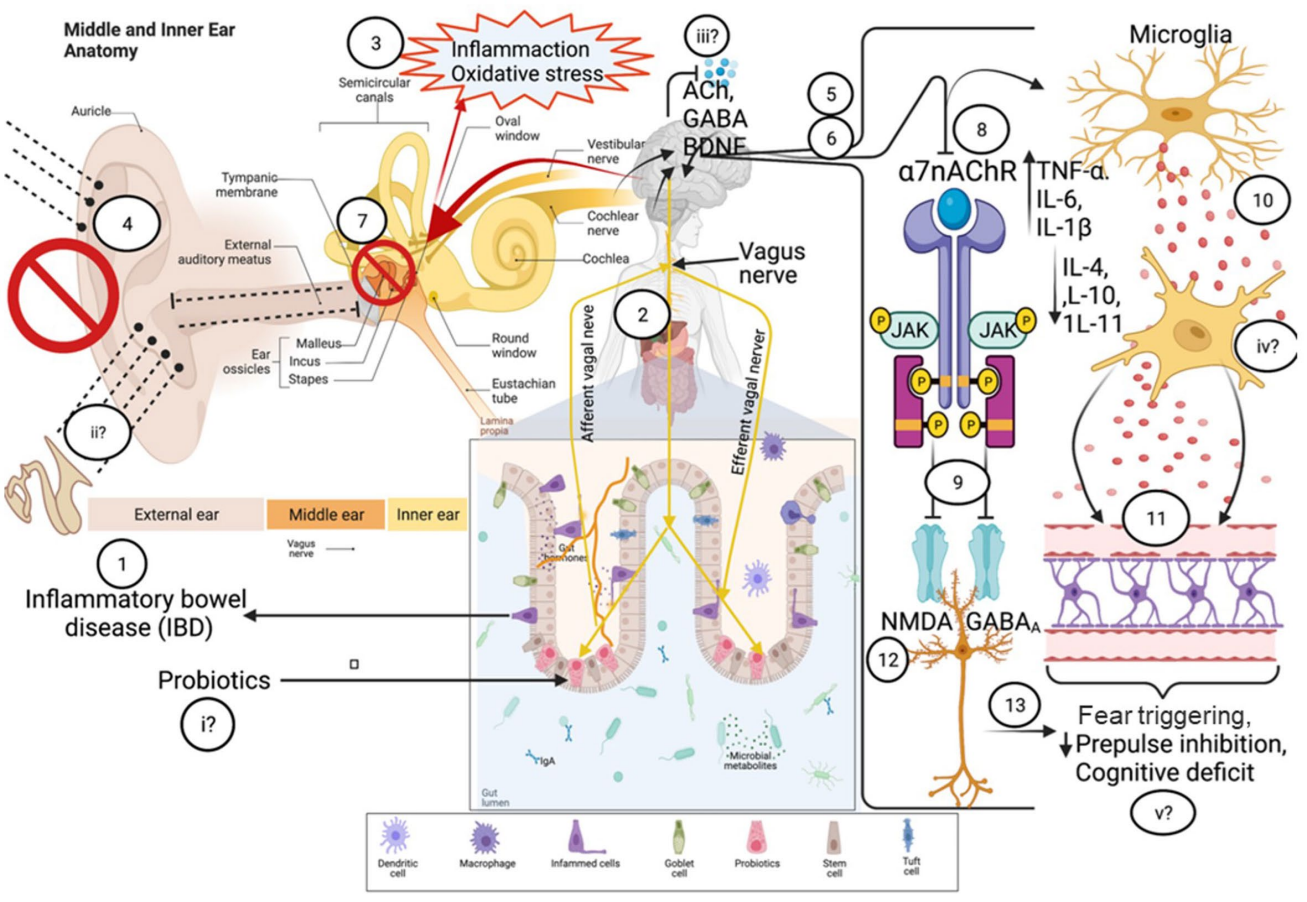
Proposed auditory-gut-brain axis: Proposed schematic flow of the auditory-gut-brain axis and its implication in gut dysfunction and probiotic intervention: (1) Dysfunctional gut system (e.g., inflammatory bowel disease, IBD) decreases vagal tone (2), increased inflammatory responses and oxidative stress in the middle ear disrupt the central auditory system and could impact the ability to process intricate auditory signals (3), resulting in sensorimotor gating impairment (4). Consequently, dysbiosis-mediated reduction of vagal tone and degraded evoked responses to acoustic stimuli due to altered processing of mis-matches in the ear would cause decreased gut-brain synthesis of neurochemical (e.g., ACh, GABA) (5) and trophic support (6), leading to altered intracellular calcium homeostasis, electro-motility and neurochemical signals between the ear and the brain thereby causing acoustic injury and loss of hair follicles and neuron survival of the inner ear (7). In the process, loss of α-7nAChR subunit or decreased ligand binding (8) induces alterations to GABA and NMDA receptor subunits in the brain (9), accompanied by astrocytic and microglia reactivity (10), increased release of pro-inflammatory cytokines and permeability of blood brain barrier (11) as well as brain region-dependent dysfunction including degeneration of hippocampal pyramidal neurons and loss of glutamatergic and GABAergic receptors of spiral ganglion neurons in the cochlea (12). This can lead to anxiety, reduced prepulse inhibition of the acoustic startle reflex, and cognitive decline (13). However, the hypothetical intervention of hearing loss with probiotics (i) is proposed to normalize hearing (ii), gut-brain-mediated release of neurotransmitters (iii), microglial physiological functions (iv) as well as behavior (v). ACh, Acetylcholine; GABA, Gamma aminobutyric acid; BDNF, Brain derived neurotrophic factor; α-7nAChR, Alpha-7 nicotinic acetylcholine receptor; NMDA, N-methyl-D-aspartate receptor; TNF-α, Tumor necrosis factor-alpha; IL-1β, Interleukin-1beta; IL-4, Interleukin-4; IL-6, Interleukin-6; IL-11, Interleukin-11. Created with BioRender.com.

In health and disease states the auditory system may be regulated, in part, by the gut-brain axis through various processes (Figure 6). However, we still have so much to understand about the cellular and molecular mechanisms underlying these relationships. Therefore, we recommend conducting future research involving single-hit or double-hit factor models to help clarify the role of gut disorders and certain neuropsychiatric or neurodegenerative diseases. Particularly, this might aid in understanding the combined roles of gut and neurological disorders in hearing loss or in sensorimotor gating mechanism-derived alterations in sound processing. For example, single-hit models could focus on direct vagal innervation received by the auditory system, while double-hit models might examine the impact of the interactions of the microbiome, vagus nerve and brain on the auditory system.

It is still unclear how vagal nerve modulation or gut-derived probiotic enrichment can lead to improved hearing in cases of auditory loss. A better understanding would provide new areas of investigation for the identification of future drug targets. The neuro-immune system, which plays a vital defence role in health and disease states, allows for hearing loss interventions through inflammatory mediators and their modulation of extracellular channels. The density of gut flora and the vulnerability of the auditory-brain system to infection or HPA stress may also be contributory factors. Specific mechanisms of sound-derived microglial reactivity, which may act as an audio-immune-surveillance cell via a gut-brain-nicotinic-cholinergic pathway, are not fully understood and would also open up new avenues for drug targets. The suggested research areas have the potential to reveal new concepts for modification, inhibition and stimulation of the auditory-gut-brain axis across a wide range of diseases linked to neurocognition and hearing impairments.

**Figure 7:**
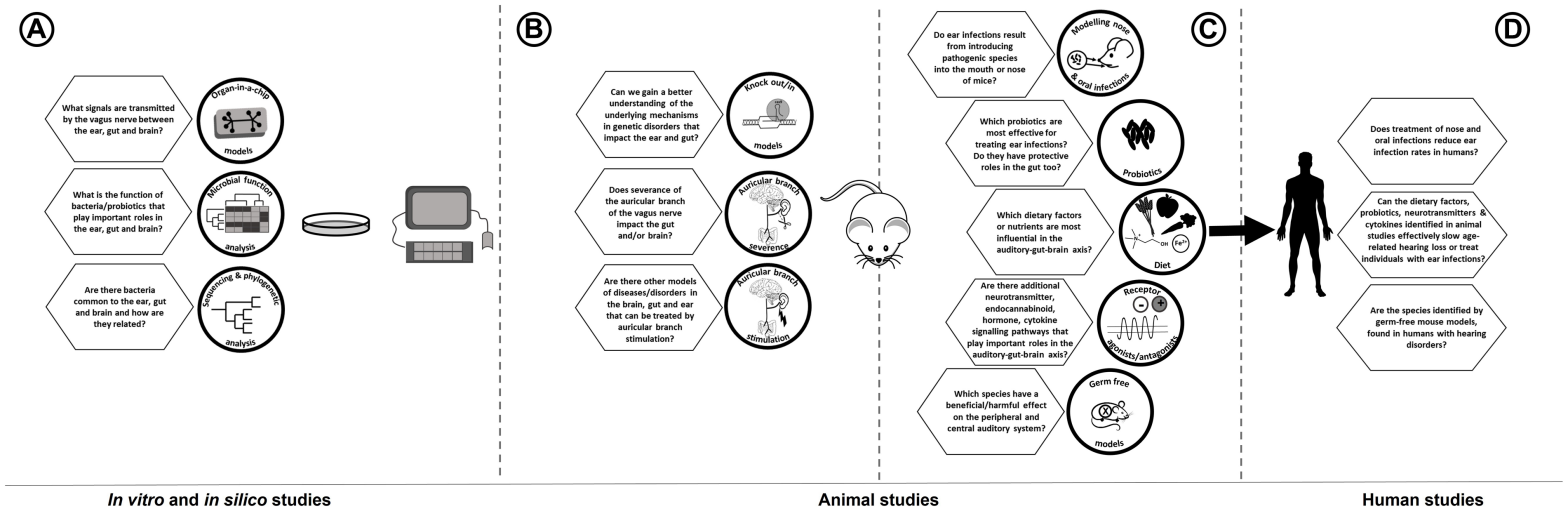
A summary of potential questions about the auditory-gut-brain axis that could be addressed in the future through **(A)** *in vitro* and *in silico* studies, **(B)** *in vivo* animal studies alone, **(C)** animal studies which can guide human studies and **(D)** human studies. We make a distinction in animal studies as some investigations with animals cannot be translated into human studies. Components of this figure used the following online resources which all have Creative Commons CC0: https://openclipart.org/detail/35791/brain-01 by rejon; https://openclipart.org/detail/269019/ear by CCX; https://www.needpix.com/photo/169098/intestines-bowel-guts-intestinal-gastrointestinal-digestive-system-abdominal-biology-science. https://openclipart.org/detail/17622/simple-cartoon-mouse-1 by lemmling; https://openclipart.org/detail/182185/man-shape by Onsemeliot.

*In vitro* research and animal models could be used in the future to investigate the key players identified in this review in greater depth, and to answer questions about the causal roles of these factors in the auditory-gut-brain axis (Figure 7). The knowledge gained from animal research could assist in guiding future research done in humans. In turn this could aid in developing targeted interventions within the context of the auditory-gut-brain axis, for addressing the various types of hearing loss, auditory-related disorders and neuropsychiatric disease, or treating ear infections.

Finally, there are many other dietary factors (e.g., omega 3 PUFAs), other signaling molecules (e.g., amino acids, sex hormones), medications/drugs and other health-related factors (e.g., physical exercise) that could be considered in future for their potential involvements in the auditory-gut-brain axis.

## 6. Conclusion

Numerous mechanisms have been implicated in the gut-brain axis and are being investigated in depth. However, little consideration has been given to the relationships that these mechanisms provide to other parts of the body. Investigation of the broader networks of signaling pathways that connect the gut-brain axis with other body parts could widen the research questions relevant to the field – ultimately assisting in the study of diseases and possible interventions.

As hearing loss poses a significant problem across the globe, irrespective of age, the auditory system is an important focus of research. There are various complex underlying causes of hearing loss, making this a challenging issue to address. In this review we have discussed research studies that provide considerable evidence of a link between the ear, gut and brain. These potential mechanisms are often interconnected, making it difficult to identify their independent roles in connecting these systems. The existing papers also span across animal and human research. However, despite these challenges, there appear to be numerous possible auditory-gut-brain axis connections.

Different levels of certainty may be placed in the putative mechanisms outlined in this review. The vagus nerve provides a direct anatomical connection between the ear, gut and brain and, as such, we can be highly confident that this is a mechanism which connects the auditory-gut-brain axis. However, although indirect roles of the immune system or extracellular signaling mechanisms have been established, further investigation is required to determine whether they directly contribute to the auditory-gut-brain axis.

## Author contributions

AG, MH, and MK outlined the review and researched literature. AG wrote the review and created most figures, while M-ÈT and BB-A wrote a perspective section and provided Figure 6. All authors assisted with reading and editing the review.

## Funding

This work has been supported in part by the National Research Foundation of South Africa (Grant Number: MND200610529926, NRF Postgraduate Scholarships) and by the National Institutes of Health (NIH) [Fogarty International Center (FIC) and National Institute of Child Health and Human Development (NICHD) R01HD093578 and R01HD085813].

## Conflict of interest

The authors declare that the research was conducted in the absence of any commercial or financial relationships that could be construed as a potential conflict of interest.

## Publisher’s note

All claims expressed in this article are solely those of the authors and do not necessarily represent those of their affiliated organizations, or those of the publisher, the editors and the reviewers. Any product that may be evaluated in this article, or claim that may be made by its manufacturer, is not guaranteed or endorsed by the publisher.
